# Directed Evolution of a Cp*Rh^III^‐Linked Biohybrid Catalyst Based on a Screening Platform with Affinity Purification

**DOI:** 10.1002/cbic.202000681

**Published:** 2020-11-09

**Authors:** Shunsuke Kato, Akira Onoda, Naomasa Taniguchi, Ulrich Schwaneberg, Takashi Hayashi

**Affiliations:** ^1^ Department of Applied Chemistry Graduate School of Engineering Osaka University 2-1 Yamadaoka Suita Osaka 565-0871 Japan; ^2^ Faculty of Environmental Earth Science Hokkaido University North 10 West 5, Kita-ku Sapporo Hokkaido 060-0810 Japan; ^3^ Institute of Biotechnology RWTH Aachen University Worringerweg 3 Aachen 52074 Germany

**Keywords:** artificial metalloenzymes, biohybrid catalysts, C−H bond functionalization, directed evolution, rhodium

## Abstract

Directed evolution of Cp*Rh^III^‐linked nitrobindin (NB), a biohybrid catalyst, was performed based on an *in vitro* screening approach. A key aspect of this effort was the establishment of a high‐throughput screening (HTS) platform that involves an affinity purification step employing a starch‐agarose resin for a maltose binding protein (MBP) tag. The HTS platform enables efficient preparation of the purified MBP‐tagged biohybrid catalysts in a 96‐well format and eliminates background influence of the host *E. coli* cells. Three rounds of directed evolution and screening of more than 4000 clones yielded a Cp*Rh^III^‐linked NB(T98H/L100K/K127E) variant with a 4.9‐fold enhanced activity for the cycloaddition of acetophenone oximes with alkynes. It is confirmed that this HTS platform for directed evolution provides an efficient strategy for generating highly active biohybrid catalysts incorporating a synthetic metal cofactor.

## Graphical abstract

**More accurate library screening**: A new high‐throughput screening platform including affinity purification has been developed based on an *in vitro* screening approach for MBP‐tagged nitrobindin having a Cp*Rh^III^ complex as an artificial cofactor. Using this platform for directed evolution, a biohybrid catalyst with clearly enhanced activity toward the cycloaddition of acetophenone oximes with alkynes was obtained.

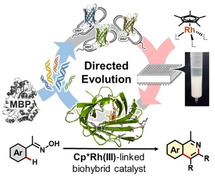

## Introduction

Incorporation of a synthetic metal cofactor within a protein cavity generates a new class of catalysts, referred to as biohybrid catalysts or artificial metalloenzymes.^[1]^ Unlike a series of conventional transition metal catalysts, biohybrid catalysts with an active site including a coordination sphere can be systematically optimized by genetic engineering.^[2]^ Directed evolution has received significant attention as a powerful strategy for engineering the protein scaffold of biohybrid catalysts.^[3]^ Through the iterative cycles of random mutagenesis and library screening, directed evolutions explore the amino acid sequence space that natures offer and have therefore a great potential to improve the performance of biohybrid catalysts. Here, we report an investigation of catalytic activity enhancement of a Cp*Rh^III^‐linked biohybrid catalyst by directed evolution.

**Figure 1:**
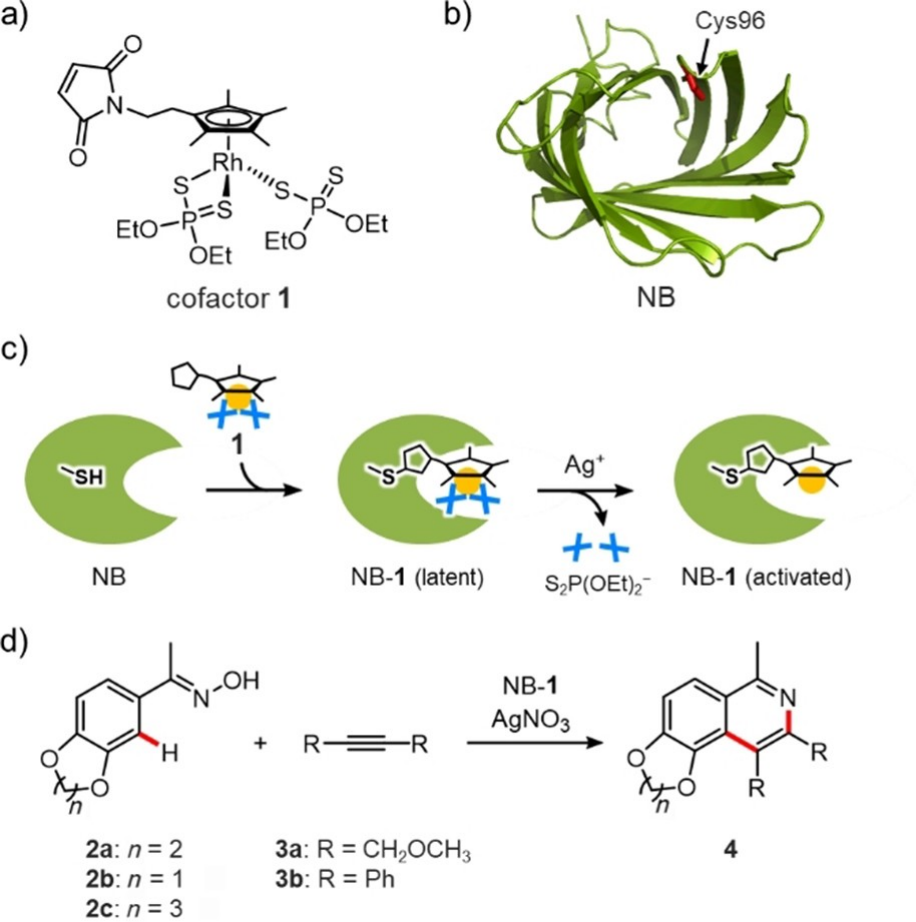
a) Chemical structure of cofactor **1**. To prevent undesirable side‐reactions of the rhodium metal center with nucleophiles, three coordination sites are protected by dithiophosphate ligands. b) Crystal structure of NB (PDB ID: 3WJB). The position of Cys96 for anchoring of the cofactor is indicated by a red stick. c) Construction of Cp*Rh^III^‐linked biohybrid catalyst (NB‐**1**). Cofactor **1** was first conjugated within the NB scaffold via a Cys‐maleimide linkage. The modified protein was subsequently activated upon addition of Ag^+^ ion to deprotect the dithiophosphate ligands. d) Cycloaddition of acetophenone oxime **2** with alkyne **3**.

Biohybrid catalysts incorporating a synthetic Cp*Rh^III^ complex as a cofactor have significant potential to enable a broad range of abiotic C−H bond functionalizations.[^4^, ^5^] Our group has recently prepared a biohybrid catalyst incorporating a Cp*Rh^III^ cofactor **1** within a hydrophobic cavity of nitrobindin (NB) protein (Figure 1a and b).^[6]^ Protection of a reactive rhodium center using dithiophosphate ligands enables us to incorporate a highly electrophilic Cp*Rh^III^ complex at a defined position within NB (Figure 1c). To further improve its activity toward the cycloaddition reaction of acetophenone oximes with alkynes via C(sp^2^)−H bond activation (Figure 1d), a directed evolution campaign of the Cp*Rh^III^‐linked biohybrid catalyst was performed.

## Results and Discussion

### Development of a high‐throughput screening platform

A crucial prerequisite for directed evolution is to establish a reliable platform for high‐throughput screening (HTS).^[7]^ In contrast to well‐established HTS for natural enzymes, HTS for biohybrid catalysts is complicated by an additional requirement to incorporate a synthetic metal cofactor.^[8]^ In the last few years, several excellent HTS platforms have been developed for biohybrid catalysts based on two major approaches using *in vivo* and *in vitro* screenings.[^3d^, ^3e^, ^8^] The *in vivo* screening platforms have directly used periplasmic^[9]^ and surface‐displayed^[10]^ protein scaffolds, thereby dramatically increasing the throughput of the directed evolution. However, such cell‐dependent systems may suffer from deactivation of the metal cofactor^[11]^ and background catalysis derived from a free metal cofactor that was not conjugated with the target protein scaffold.^[12]^ The screening in cell lysates has the same problems.^[13]^ Therefore, the *in vitro* screening platform using a purified biohybrid catalyst will be a promising and general strategy for directed evolution of biohybrid catalysts.[^13a^, ^14^] The main challenge of this strategy is to improve throughput of the screening. As conventional protein purification methods are time‐ and cost‐intensive, the screened library size in previous reports has been limited to between a few dozen and a few hundred clones.[^13a^, ^14^]

To address this challenge associated with *in vitro* screening, we have focused on an affinity purification system which uses a maltose binding protein (MBP) tag.^[15]^ Compared to the other standard purification tags, the use of the MBP tag provides several advantages. One main advantage is that the MBP‐tagged proteins can be purified in a facile one‐step method using a custom‐designed starch‐agarose resin (Figure S1 in the Supporting Information). It is possible to easily prepare the starch‐agarose resin on a laboratory scale from inexpensive materials using a modified procedure (Table S1).^[16]^ Another advantage is that the MBP tag increases the solubility of a fusion partner protein, which should ensure its general applicability and construction of a biohybrid catalyst library with high quality (Figure S2).^[17]^ Furthermore, the maltose solution used in the elution step does not interfere with the catalysis of the Cp*Rh^III^ cofactor (Figure S3). Therefore, a removal of maltose is not required before screening.

**Figure 2:**
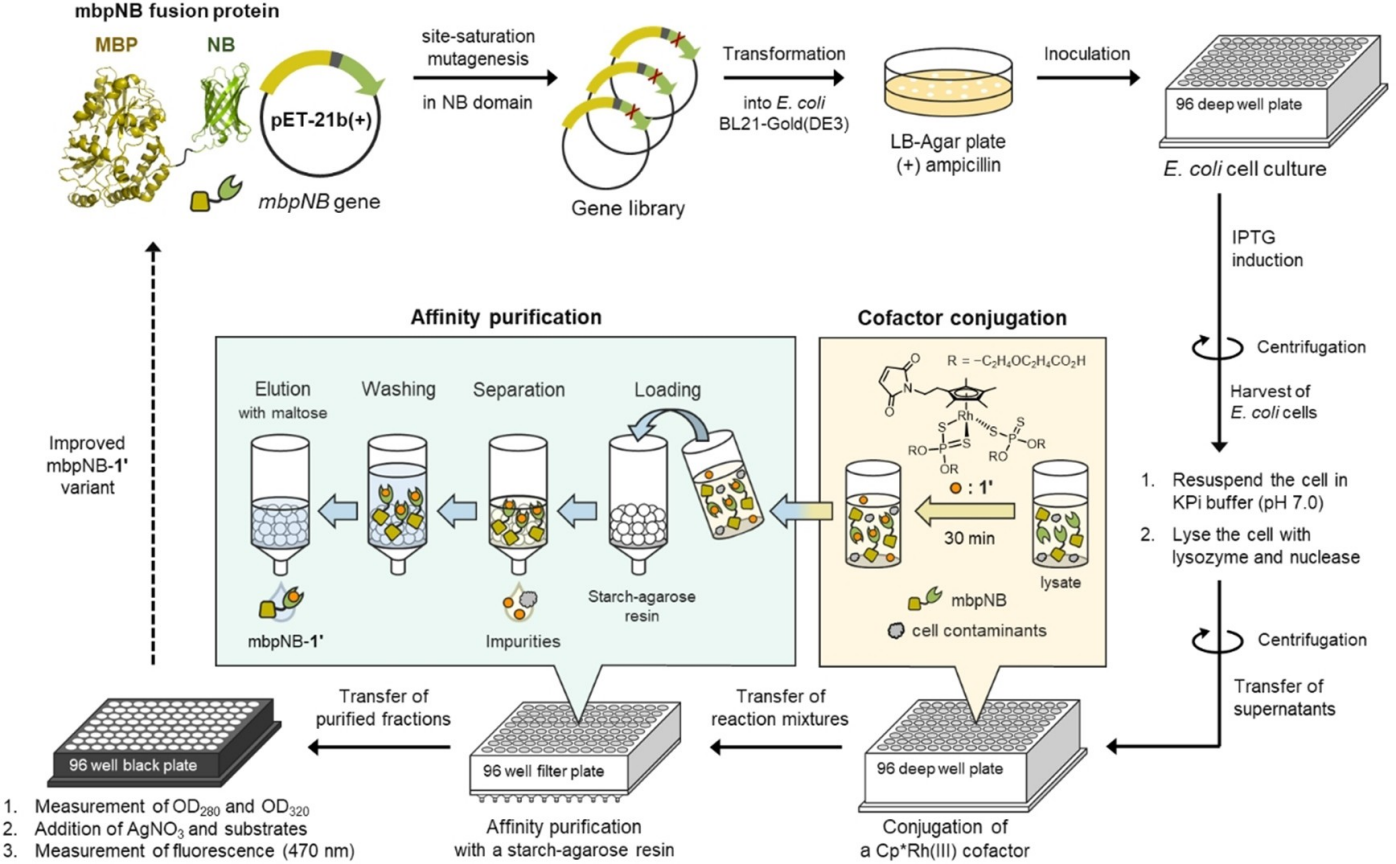
Screening workflow for directed evolution of the Cp*Rh^III^‐linked biohybrid catalyst. The four main steps of the HTS platform include: expression of mbpNB fusion protein, cofactor conjugation in a cell lysate, affinity purification using the starch‐agarose resin, and evaluation in a 96‐well format.

With knowledge of these advantages provided by the MBP tag, we established a new HTS platform for directed evolution of the Cp*Rh^III^‐linked biohybrid catalyst (Figure 2). A fusion protein designated mbpNB, in which the MBP tag is connected to the *N*‐terminus of NB, was first prepared. Starting from the *mbpNB* constructs in a pET‐21b(+) vector, selected residues of the NB domain were randomized by site‐saturation mutagenesis (SSM). The resulting library of mbpNB was expressed in *E. coli* cells in a 96‐well format. *E. coli* cells harboring mbpNB variants were then lysed, and the mbpNB variants in supernatant were directly subjected to conjugation with an excess amount of Cp*Rh^III^ cofactor **1**′ having two *O,O′*‐dicarboxyethoxyethyl dithiophosphate ligands.^[18]^ The reaction mixture was then loaded onto a 96‐well filter plate, which was packed with starch‐agarose resin, in order to purify the **1**′‐linked mbpNB (mbpNB‐**1**′). The resin was subsequently washed to remove contaminants from *E. coli* cells and free cofactor molecules that were not conjugated with mbpNB scaffold. Finally, mbpNB‐**1**′ was eluted with a maltose solution to obtain a library of purified biohybrid catalysts. The formation of mbpNB‐**1**′ and its purity were confirmed by SDS‐PAGE, UV/Vis, and ESI‐TOF MS analyses (Figures S4–S7). This purification system allows us to quickly estimate the concentration of mbpNB‐**1**′ by measuring its absorption at 320 nm (OD_320_) derived from the cofactor **1**′.

Activity screening of the prepared mbpNB‐**1**′ variants was performed using a fluorescence microplate reader. The mbpNB‐**1**′ in the purified fraction was activated upon addition of AgNO_3_, and subjected to screening through the cycloaddition reaction of 3′,4′‐ethylenedioxyacetophenone oxime (**2 a**) with 1,4‐dimethoxy‐2‐butyne (**3 a**).^[19]^ The product yields were determined from the fluorescence intensity at 470 nm (*FI*_470_) derived from the isoquinoline product **4 aa**. The activity was determined from the product yield divided by the concentration of the cofactor (*FI*_470_/OD_320_). It is noted that this HTS platform completely excludes the background reaction catalyzed by free cofactor **1**′ that is not conjugated with mbpNB scaffold (Figure S8). A negative control using an *E. coli* cell lysate without expressed mbpNB does not produce a fluorescence signal. This indicates that the catalytic activity of mbpNB‐**1**′ can be accurately determined.

### Directed evolution of a Cp*Rh^III^‐linked biohybrid catalyst

**Figure 3:**
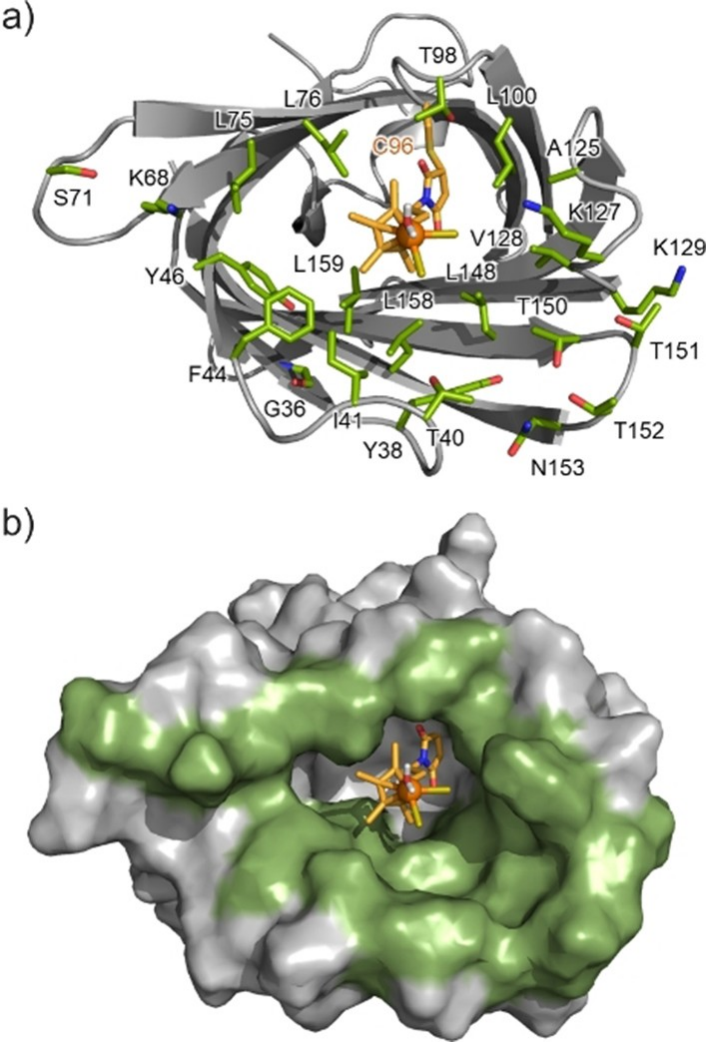
MD structure of Cp*Rh^III^‐linked NB with a) cartoon and sticks and b) modeled surfaces. The MD simulation was performed based on the crystal structure of NB (PDB ID: 3WJB). The dithiophosphate ligands of cofactor **1** were substituted with two chloride ions and one water molecule. The 23 residues subjected to SSM are highlighted in green.

We then started the directed evolution of the Cp*Rh^III^‐linked biohybrid catalyst using the developed HTS platform. In the first round of the evolution, twenty‐three NB‐positions (G36, Y38, T40, I41, F44, Y46, K68, S71, L75, L76, T98, L100, A125, K127, V128, K129, L148, T150, T151, T152, N153, L158, and L159) that would function as the first, second, or outer coordination sphere of the Cp*Rh^III^ cofactor were selected for SSM (Figure 3a). These prospective residues are located in the β‐strands and loop regions which form the hydrophobic cavity of NB (Figure 3b and S9).[^20^, ^21^] These residues were randomized using the QuikChange protocol with a mixture of three oligonucleotide primers including NHN, VNN and TGG codons that do not encode for a Cys residue because the Cys substitution would provide an additional conjugation site for cofactor **1**′ (Table S2).^[22]^

After screening of more than 2000 clones in the first round of directed evolution, we selected ten Cp*Rh^III^‐linked mbpNB variants as a candidate and screened these variants again to identify the hits of the first generation (Figure S10). In particular, two mbpNB‐**1**′ variants, T98H and N153I, have clearly higher activity, as scored by the *FI*_470_/OD_320_ values, relative to the parent mbpNB‐**1**′. However, the mbpNB(N153I)‐**1**′ variant was obtained from the *E. coli* expression medium at a low concentration according to OD_280_ value. Judging from the *FI*_470_/OD_320_ and OD_280_ values, mbpNB(T98H)‐**1**′ was finally selected for the next round of evolution.

**Figure 4:**
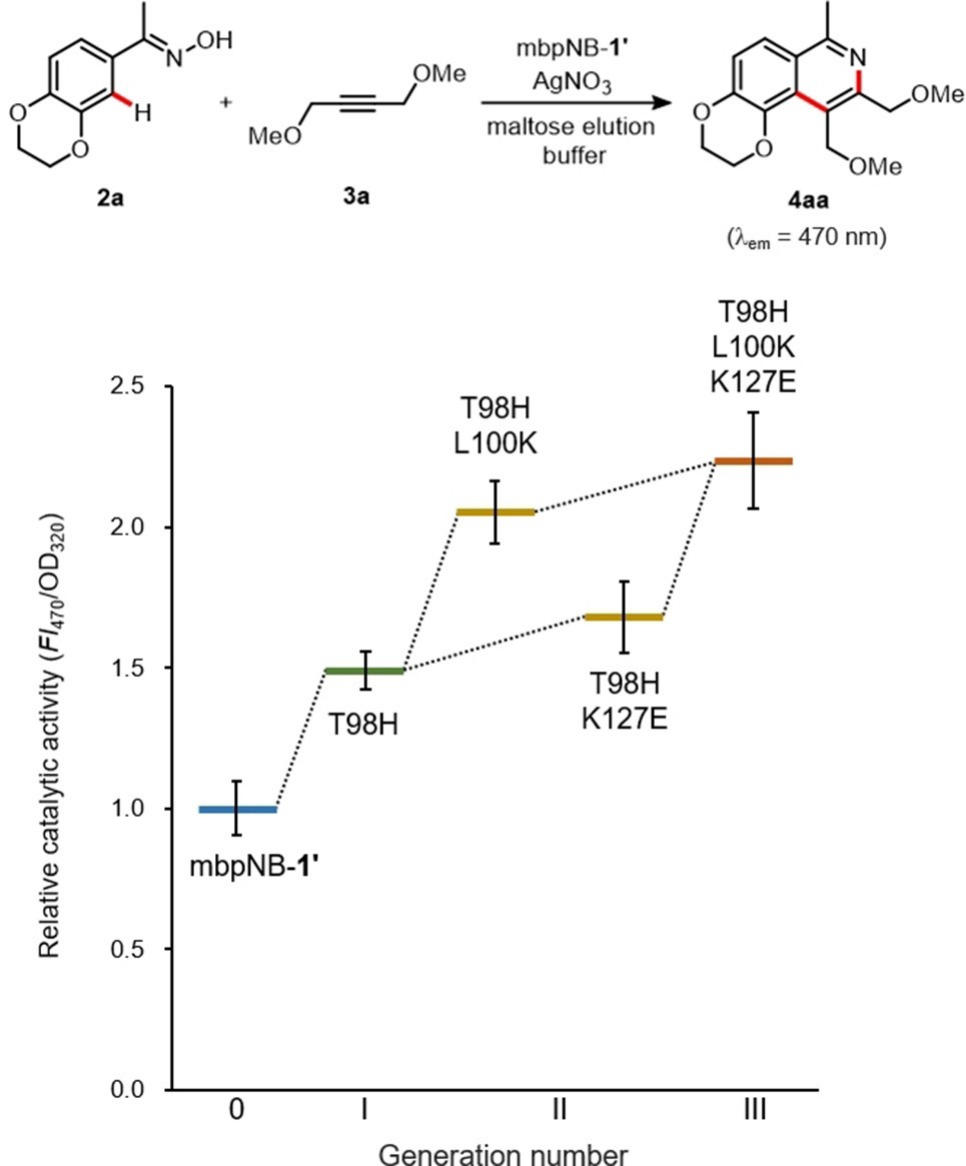
Cycloaddition of **2 a** with **3 a** catalyzed by evolved mbpNB‐**1**′ variants. Reaction conditions: **2 a** (0.125 mM), **3 a** (2.0 mM) and AgNO_3_ (1.0 mM) in a maltose elution buffer (25 mM maltose, 100 mM AcOH, 50 mM MES, 2 % 1,4‐dioxane, pH 5.5) containing mbpNB‐**1**′ variants (ca. 2 μM), 37 °C for 72 h.

In the second round, SSM of mbpNB(T98H)‐**1**′ was performed targeting ten amino acid residues (G36, Y38, F44, Y46, S71, L100, A125, K127, K129, and N153) which were selected based on structural consideration and screening of the SSM libraries. After screening of more than 1000 clones, we identified two promising variants, mbpNB(T98H/L100K)‐**1**′ and mbpNB(T98H/K127E)‐**1**′ (Figure 4). Lys100 and Glu127 residues were expected to be located in the opposite side of the rhodium metal center according to the MD simulation (Figure S11). Based on this finding, we carried out a third round focusing on these two additional “hot spot” residues. SSM of mbpNB(T98H/K127E)‐**1**′ was performed targeting the L100 residue, and then we discovered the mbpNB(T98H/L100K/K127E)‐**1**′ variant as the best performing biohybrid catalyst. These results indicate that two key substitutions, L100→K100 and K127→E127, enhance the activity in a cooperative manner. mbpNB(T98H/L100K/K127E)‐**1**′ with the three substitutions has a 2.2‐fold increase in catalytic activity (Figure 4 and S12).

### Evaluation of the biohybrid catalysts

After three rounds of directed evolution, we identified three key positions (T98→H98, L100→K100 and K127→E127) that modulate the cycloaddition activity of the Cp*Rh^III^ cofactor. To further evaluate the contribution of the substitutions, we prepared three NB variants, NB(T98H), NB(T98H/L100K), and NB(T98H/L100K/K127E) without the MBP tag. These variants were expressed using a pET‐42b(+) plasmid with a strep‐tag gene, and purified using a Strep‐Tactin column (Figure S13). CD measurements indicated that these three NB variants maintain β‐barrel structures similar to those of the original NB (Figure S14).^[21]^ The NB variants were then conjugated with cofactor **1** to prepare biohybrid catalysts, NB(T98H)‐**1**, NB(T98H/L100K)‐**1**, and NB(T98H/L100K/K127E)‐**1**. The conjugation reaction was found to proceed efficiently for all three NB variants, and formation of each biohybrid catalyst was confirmed by MALDI‐TOF MS (Figure S15).

**Figure 5:**
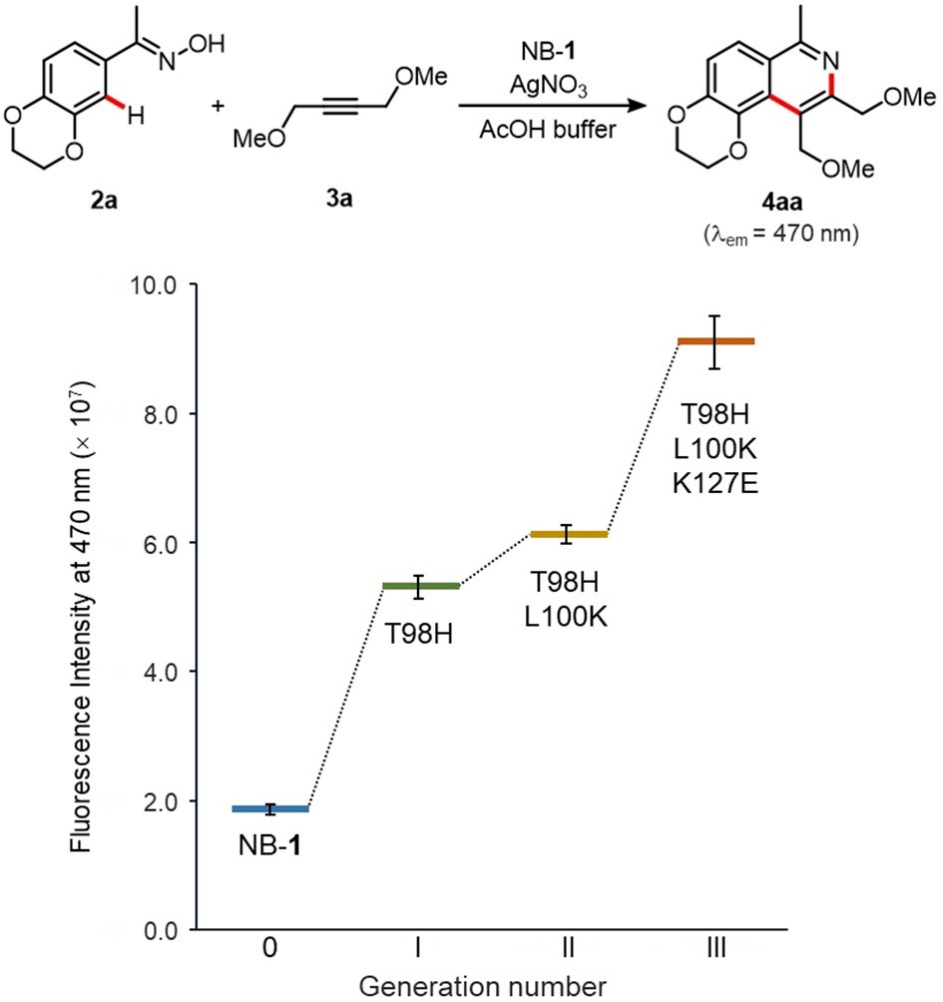
Cycloaddition of **2 a** with alkyne **3 a** catalyzed by evolved NB‐**1** variants. Reaction conditions: NB‐**1** variant (20 μM), **2 a** (0.125 mM), **3 a** (2.0 mM) and AgNO_3_ (0.1 mM) in AcOH buffer (100 mM AcOH, 2 % 1,4‐dioxane, pH 4.0), 25 °C, 48 h.

The prepared biohybrid catalysts were then investigated for the cycloaddition reaction of **2 a** with **3 a**. The reactions were performed under the optimized conditions according to our previous study (20 μM catalyst in AcOH buffer at pH 4.0),^[6]^ and it was found that all three biohybrid catalyst variants had enhanced catalytic activities for the cycloaddition reaction (Figure 5). In particular, NB(T98H/L100K/K127E)‐**1** was found to provide product **4 aa** with a 4.9‐fold increase in activity compared to the original NB‐**1**. Formation of compound **4 aa** was also confirmed by GC‐MS (Table S3). Moreover, the activity of each NB‐**1** variant shows a clear correlation relative to that obtained by the mbpNB‐**1**′ variants as shown in Figure 4. These results prove the reliability of our MBP‐based HTS method for directed evolution of biohybrid catalysts.

**Figure 6:**
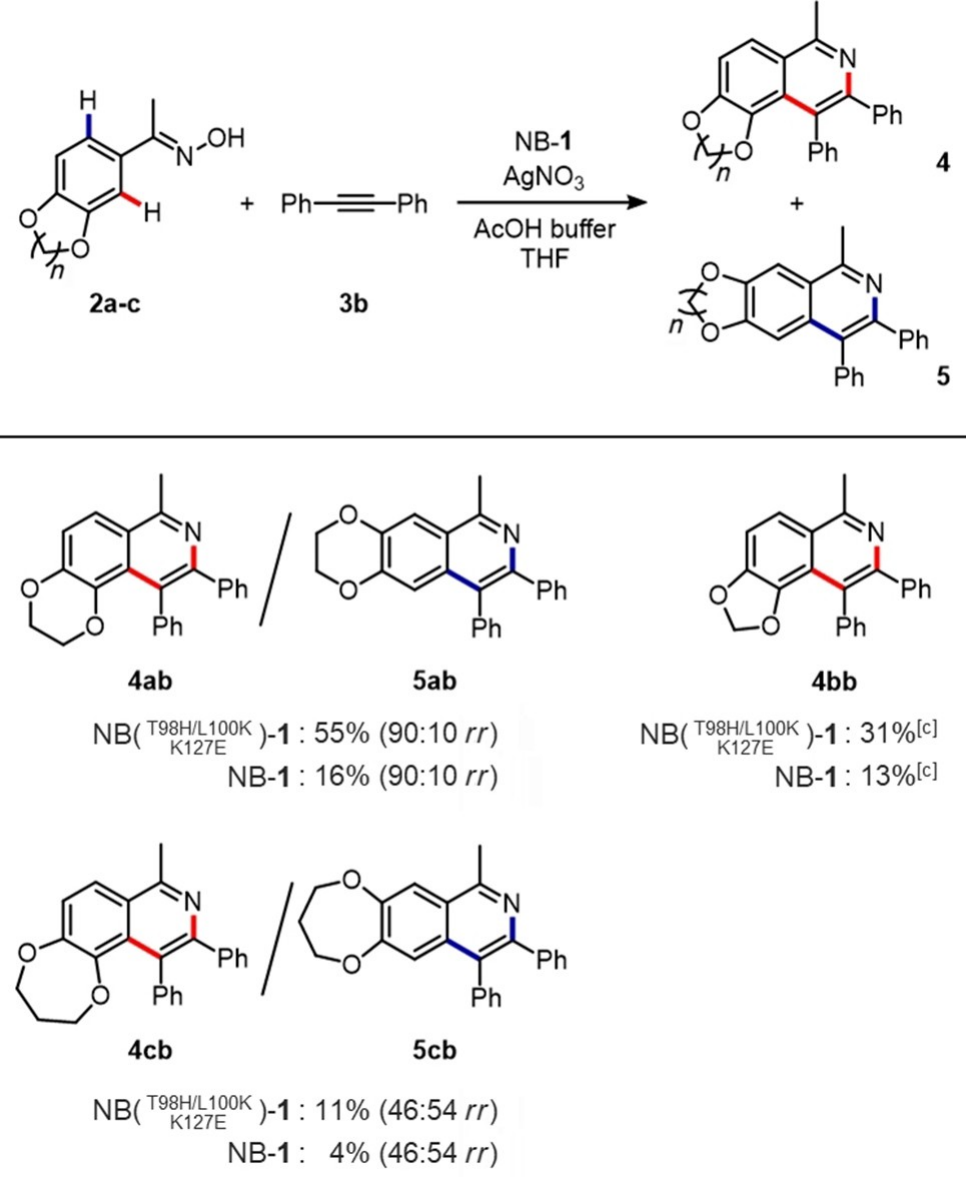
Cycloaddition of **2 a**–**c** with diphenylacetylene **3 b** catalyzed by NB‐**1** and NB(T98H/L100K/K127E)‐**1**. Reaction conditions: NB‐**1** variant (20 μM), **2 a**–**c** (0.125 mM), **3 b** (2.0 mM) and AgNO_3_ (1.0 mM) in AcOH buffer (100 mM AcOH, 20 % THF, pH 4.0), 25 °C, 120 h. Total yields of **4** and **5** and regioisomeric ratio (*rr*) were determined by GC‐MS. (A trace amount of regioisomer **5 bb** was generated: <1.0 % yield).

NB(T98H/L100K/K127E)‐**1** was also found to exhibit improved activity toward the cycloaddition of acetophenone oximes **2 a**–**c** with diphenylacetylene **3 b** (Figure 6). In the reaction of **2 a** with **3 b**, NB(T98H/L100K/K127E)‐**1** provided **4 ab** and **5 ab** in 55 % total yield with a 3.4‐fold increase in activity compared to the original biohybrid catalyst NB‐**1**. NB(T98H/L100K/K127E)‐**1** also shows higher activity toward the cycloaddition reactions with substrates **2 b** and **2 c** which differ in the ring size of catechol ether. The regioisomeric ratios of products **4** and **5** did not change between NB(T98H/L100K/K127E)‐**1** and NB‐**1** in all cases for the substrates **2 a**–**c**. This finding indicates that the His98, Lys100 and Glu127 residues do not contribute to substrate binding. It is believed that these three mutated residues favorably interact with the Cp*Rh^III^ cofactor **1** and/or Ag^+^ ions involved in the exchange of the dithiophosphate ligands, thereby improving the activity toward the cycloaddition reactions.

## Conclusion

In summary, we have generated Cp*Rh^III^‐linked biohybrid catalysts having enhanced reactivity toward the C(sp^2^)−H bond functionalization by directed evolution using one of the first developed and validated HTS platforms for biohybrid catalysts. This HTS platform, which includes affinity purification using an MBP tag, allows us to efficiently eliminate the contaminants from *E. coli* cells and the background catalysis derived from the free metal cofactor, thereby improving the accuracy of library screening in the 96‐well format. Therefore, this HTS platform provides a versatile and powerful system for directed evolution of biohybrid catalysts that can likely be applied to a broad range of protein scaffolds and metal cofactors. Through three rounds of directed evolution, more than 4000 clones were screened by using this HTS platform, and the promising NB(T98H/L100K/K127E)‐**1** variant was identified. This variant was found to exhibit a 4.9‐fold increase relative to the original catalyst in terms of catalytic activity toward cycloaddition reactions of acetophenone oximes with alkynes.

## Experimental Section

**Preparation of starch‐agarose resin**: Starch‐agarose resin was prepared according to the literature^[16a]^ with several modifications to generate micro‐sized particles based on the emulsification technique.^[16b]^ Agarose (11.2 g) and soluble starch (4.2 g) were added into an aqueous NaCl solution (280 mL, 0.9 wt%), and the solution was heated at 80 °C for approximately 30 min. After agarose and soluble starch were completely dissolved, paraffin oil (490 mL) containing tween 80 (3 wt%) preheated at 80 °C was added into the solution, and the mixed solution was vigorously stirred to form a water‐in‐oil (W/O) emulsion. The mixed solution of the W/O emulsion was then chilled on an ice bath with stirring to solidify the water phase of the droplets containing agarose. After 30 min, EtOH (400 mL) was added and the resulting mixture was then centrifuged at 1500 *g* for 5 min, and the supernatant was discarded. The precipitates of starch‐agarose resins were then resuspended in H_2_O, and centrifuged again at 1500 *g* for 5 min. This washing step was repeated until the paraffin oil phase was completely removed from the resins. The starch‐agarose resins were finally resuspended in KPi buffer (20 mM KPi, 200 mM NaCl, 20 *v*/*v*% EtOH, pH 7.0), and stored at 4 °C until use.

**Subcloning of expression plasmids for mbpNB**: The expression plasmid for mbpNB (a fusion protein of MBP and NB via an α‐helix linker) was constructed in two steps according to the standard subcloning protocol of NEBuilder HiFi DNA Assembly cloning kit (New England Biolabs Japan). First, a gene for MBP was inserted into a pET‐21b(+) vector encoding an NB gene. An insert encoding an MBP gene was amplified by PCR using a pMAL‐c5x plasmid (New England Biolabs Japan) as a template. The PCR products were then treated with *Dpn*I restriction enzymes (New England Biolabs Japan), purified by agarose gel electrophoresis, and assembled with a linearized pET‐21b(+) vector encoding the NB gene using NEBuilder HiFi DNA Assembly. The assembled products were transformed into chemically competent *E. coli* DH5α cells to afford a plasmid encoding MBP and NB genes. Secondly, the α‐helix linker was inserted between the MBP gene and the NB gene to generate mbpNB. The pET‐21b(+) plasmid encoding MBP and NB was linearized by PCR. The PCR products were treated with DpnI restriction enzymes, and purified by agarose gel electrophoresis. The gene of the α‐helix linker was inserted to the purified linearized vector using NEBuilder HiFi DNA Assembly. The assembled product was then transformed into chemically competent *E. coli* DH5α cells to afford the expression plasmid for mbpNB with the α‐helix linker.

**Preparation of a gene library of mbpNB**: An SSM gene library of mbpNB variants was constructed according to the standard protocol of the QuikChange II site‐directed mutagenesis kit (Agilent Technologies). The template plasmids were amplified by PCR using primers that contain NHN, VNN, and TGG codons at the target position of site‐saturation mutagenesis. The PCR products were treated with DpnI restriction enzymes, and transformed into chemically competent *E. coli* DH5α cells to afford a gene library of the mbpNB variants.

**Expression of mbpNB library in 96‐well microplate**: The prepared plasmids encoding gene library of mbpNB variants were transformed into the *E. coli* BL21‐Gold(DE3) strain, and the cells were inoculated on LB agar plates supplemented with 100 μg/mL ampicillin. The single colonies were transferred into a 96‐well microplate filled with 500 μL of LB medium (100 μg/mL ampicillin), and the cells were cultivated at 37 °C and 1500 rpm overnight. A part of the overnight cultures (100 μL) in the well was transferred into another 96‐well microplate, and stored at −80 °C after the addition of 100 μL of sterile glycerol (40 *v*/*v* %). Another part of the overnight cultures (7.0 μL) was inoculated into 700 μL of LB medium (100 μg/mL ampicillin) in another 96‐well microplate, and incubated at 37 °C and 1500 rpm for 2.5 h. After 50 μL of LB medium (100 μg/mL ampicillin) containing isopropyl‐β‐_D_‐1‐thiogalactopyranoside (IPTG; 3.0 mM) was added, the incubation was continued at 18 °C and 1500 rpm for 20 h. *E. coli* cells were then harvested by centrifugation at 2200 *g* for 5 min, and stored at −20 °C until further use.

**Preparation of a purified SSM library of mbpNB‐1′ in a 96‐well microplate**: Frozen *E. coli* cells in a 96‐well microplate were thawed at room temperature for 20 min. The cells were then lysed by resuspending in 155 μL of KPi buffer (20 mM KPi, 200 mM NaCl, pH 7.0) containing lysozyme (5.0 ng) and benzonase® nuclease (1.25 U). After incubation for 1 h at 37 °C, lysed cells were centrifuged at 2200 *g* for 5 min. Supernatants were transferred to another 96‐well microplate. The resulting cell pellets were resuspended in 200 μL of KPi buffer (20 mM KPi, 200 mM NaCl, pH 7.0), and centrifuged again at 2200 *g* for 5 min to combine the supernatants. Into the combined supernatant, 200 μL of KPi buffer (20 mM KPi, 200 mM NaCl, pH 7.0) supplemented with cofactor **1**′ (188 μM) was added. After incubation for 30 min at room temperature, the reaction mixture was loaded into a 96‐well filter plate that was packed with 500 μL of starch‐agarose resin equilibrated with KPi buffer (20 mM KPi, 200 mM NaCl, pH 7.0). The resins were washed with 1) 0.9 mL of KPi buffer (20 mM KPi, 200 mM NaCl, pH 7.0), 2) 3×0.9 mL of AcOH‐MES buffer (100 mM AcOH, 50 mM MES, pH 5.5), and 3) 200 μL of AcOH‐MES buffer (100 mM AcOH, 50 mM MES, pH 5.5) containing maltose (_D_‐(+)‐maltose; 25 mM). The SSM library of mbpNB‐**1**′ was then eluted with 400 μL of AcOH‐MES buffer (100 mM AcOH, 50 mM MES, pH 5.5) containing maltose (25 mM). The concentration of the mbpNB‐**1**′ variants in the elution was estimated by UV/Vis absorption at 280 and 320 nm.

**Activity screening of a SSM library of mbpNB‐1′**: Solutions of substrate **2 a** (37.5 nmol) in 1,4‐dioxane (3 μL), **3 a** (600 nmol) in 1,4‐dioxane (3 μL), and AgNO_3_ (300 nmol) in H_2_O (6 μL) were added to the purified fractions of mbpNB‐**1**′ in 300 μL of AcOH‐MES buffer (100 mM AcOH, 50 mM MES, 25 mM maltose, pH 5.5) in a 96‐well microplate. After incubation at 37 °C for 72 h, the reaction mixtures were centrifuged at 2200 *g* for 5 min, and directly analyzed by fluorescence spectroscopy to quantify the formation of compound **4 aa** (*λ*_ex_=270 nm, *λ*_em_=470 nm).

**Subcloning of expression plasmid for NB variants with the strep‐tag II sequence**: The expression plasmids containing the NB gene with a strep‐tag II gene in the pET‐42b(+) vector were prepared according to the reported procedure.^[20]^ Other expression plasmids for NB(T98H), NB(T98H/L100K), and NB(T98H/L100K/K127E) with strep‐tag II were constructed according to the standard subcloning protocol of NEBuilder HiFi DNA Assembly cloning kit. Each insert gene of the NB variants was amplified by PCR from the pET‐21b(+) plasmid encoding mbpNB(T98H), mbpNB(T98H/L100K), and mbpNB(T98H/L100K/K127E). The vector backbone with the strep‐tag II gene was amplified by PCR using the pET‐42b(+) plasmid encoding the NB gene as a template. The PCR products were then treated with DpnI restriction enzymes, purified by agarose gel electrophoresis, and assembled by NEBuilder HiFi DNA Assembly. The assembled products were transformed into chemically competent *E. coli* DH5α cells to afford the expression plasmids for NB(T98H), NB(T98H/L100K), and NB(T98H/L100K/K127E).

**Preparation of NB‐1 variants**: The expression and purification of the NB variants with strep‐tag II was performed according to our previous report.^[20]^ The NB variants (500 μM) in 500 μL of 100 mM Tris**⋅**HCl buffer (pH 8.0) containing 1.0 mM EDTA and 150 mM NaCl were reduced with DTT (0.5 μmol), and purified with a HiTrap desalting column (GE healthcare) equilibrated with a buffer solution (50 mM MOPS, 100 mM AcONa, pH 7.0). To a solution of the NB variants (ca. 20 μM) in 10 mL of MOPS buffer (50 mM MOPS, 100 mM AcOH, pH 7.0), cofactor **1** in DMSO (25 mM, 32 μL) was added, and the reaction mixture was stored on ice for 1 h. The reaction mixture was then concentrated to a volume of 1.0 mL using an Amicon Ultra centrifugal filter Unit (Millipore), and purified using a HiTrap desalting column to afford the NB conjugate with cofactor **1** (NB‐**1**). The purified NB‐**1** variants were characterized by MALDI‐TOF MS.

**Cycloaddition of 2 a–c with 3 b catalyzed by NB‐1 variants**: Solutions of substrates **2 a**–**c** (62.5 nmol) in THF (5 μL), **3 b** (1.0 μmol) in THF (5 μL), and AgNO_3_ (0.5 μmol) in H_2_O (5 μL) were added to a solution of the NB‐**1** variants (10 nmol, final concentration: 20 μM) in 500 μL of 100 mM AcOH buffer (pH 4.0) containing 20 % THF and the reaction mixture was incubated at 25 °C for 120 h. The reaction mixture was then extracted twice with 1.0 mL of diethyl ether and the organic layer was dried *in vacuo*. The residue was dissolved in EtOAc (200 μL) containing 0.50 mM acridine as an internal standard, and the solution was analyzed by GC‐MS.

## Conflict of interest

The authors declare no conflict of interest.

## Supporting information

As a service to our authors and readers, this journal provides supporting information supplied by the authors. Such materials are peer reviewed and may be re‐organized for online delivery, but are not copy‐edited or typeset. Technical support issues arising from supporting information (other than missing files) should be addressed to the authors.

Supplementary
