# Supplementary material for: Directed Evolution of a Cp*RhIII‐Linked Biohybrid Catalyst Based on a Screening Platform with Affinity Purification
Source: Chembiochem. 2020 Nov 9;22(4):679–85. doi: 10.1002/cbic.202000681 (PMC7894531; doi:10.1002/cbic.202000681)
Supplement: Supplementary file 1 — Supplementary [file CBIC-22-679-s001.pdf]

ChemBioChem

Supporting Information

Directed Evolution of a Cp*Rh^{III}-Linked Biohybrid Catalyst Based on a Screening Platform with Affinity Purification

Shunsuke Kato, Akira Onoda,* Naomasa Taniguchi, Ulrich Schwaneberg, and Takashi Hayashi*

Supporting Information

General Information

Instruments. ^{1}H NMR, ^{13}C NMR, and ^{31}P NMR spectra were recorded on a Bruker NMR Advance III HD spectrometer (400 MHz). ^{1}H and ^{13}C NMR chemical shifts are reported relative to deuterated solvents. ^{31}P NMR chemical shifts are reported relative to 85% H_3PO_4 in D_2O (0 ppm). ESI-TOF MS analyses were performed on a Bruker micrOTOF focus III mass spectrometer. MALDI-TOF MS analyses were performed on a Bruker autoflex III mass spectrometer. The GC-MS experiments were performed on a Shimadzu GCMS-QP2010 Ultra Gas Chromatograph Mass Spectrometer. UV-vis spectra were measured with a Shimadzu UV-2700 spectrophotometer and Molecular Devices SpectraMax iD5 Multi-Mode Microplate Reader. Fluorescence spectroscopic measurements were carried out with a JASCO Spectrofluorometer FP-8600 and a Molecular Devices SpectraMax iD5 Multi-Mode Microplate Reader. CD spectra were measured using a JASCO J-820AC Spectrometer. PCR was performed using Bio-Rad T100™ Thermal Cycler. Ultrapure water was demineralized using a Merck Millipore Milli-Q integral 3 system.

Materials. Oligonucleotides were obtained from Sigma-Aldrich, Inc. Nucleotide sequences were determined by FASMAC Co., Ltd and Eurofins Scientific SE, Inc. All reagents of the highest guaranteed grade were purchased from TCI Co. Ltd., Sigma-Aldrich, Inc., FUJIFILM Wako Pure Chemical Corporation and Nacalai Tesque, Inc., and used as received unless otherwise noted. Agarose was purchased from Sigma-Aldrich, Inc. (molecular biology grade, low EEO). Soluble starch was purchased from Nacalai Tesque, Inc. (molecular biology grade). Paraffin oil was purchased from Sigma-Aldrich, Inc. (IR spectroscopy grade). Tween 80 was purchased from TCI Co. Ltd. The rhodium cofactor **1**, 3′,4′-ethylenedioxyacetophenone oxime (**2a**), and the authentic samples of isoquinolines **4ab** and **5ab** were synthesized as described in our previous report.[1] *tert*-Butyl 3-(2-hydroxyethoxy)propanoate (**S1**)[2] and $[(\eta^5\text{-}Me_4Cp(CH_2)_2NH_3)RhCl_2]_2Cl_2$ **S2**[3] were synthesized according to the literature.

Synthesis Procedure

Scheme S1. Synthetic scheme of cofactor **1′**

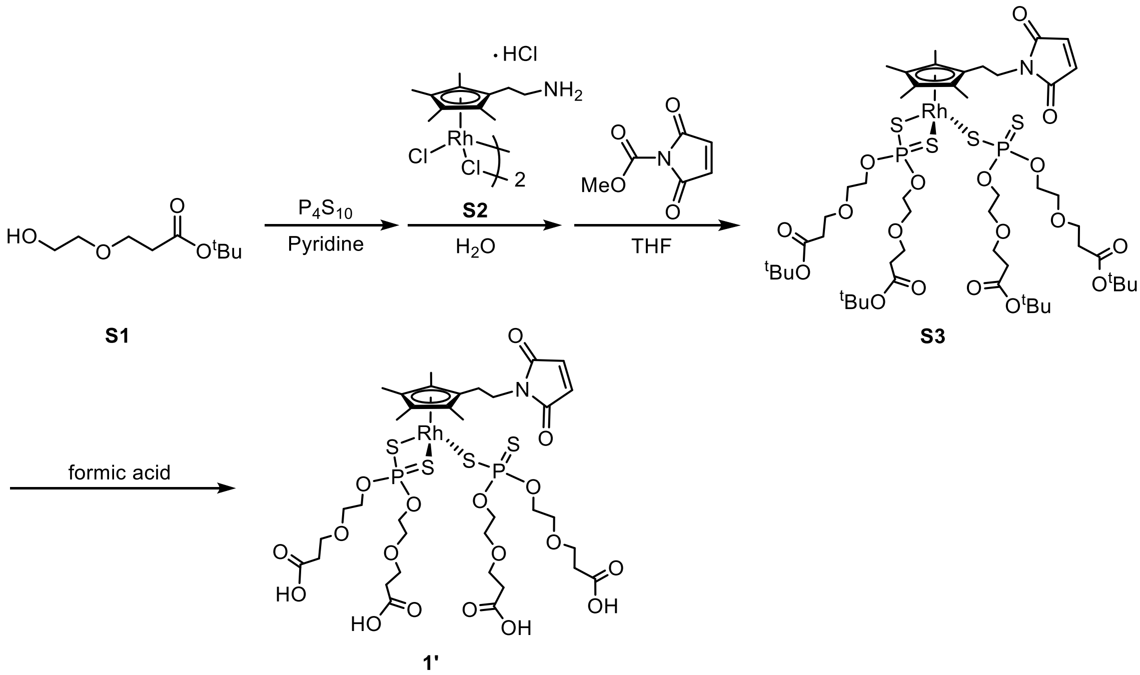

Synthesis of S3: P_4S_{10} (56.5 mg, 0.127 mmol) was dissolved in anhydrous pyridine (5 mL), and the solution was stirred for 10 min at room temperature in a glove box. *tert*-Butyl 3-(2-hydroxyethoxy)propanoate (**S1**) (163 mg, 1.02 mmol) was added into the solution at 80 °C and the reaction mixture was stirred overnight. After the reaction mixture was cooled to room temperature, generated H_2S gas was removed by N_2 bubbling. A solution containing pyridinium dithiophosphate was added into a solution of $[(\eta^5\text{-}Me_4Cp(CH_2)_2NH_3)RhCl_2]_2Cl_2$ **S2** (85.6 mg, 0.229 mmol) in H_2O (40 mL), with stirring for 5 min at room temperature. The reaction mixture was extracted with $CHCl_3$. The organic layer was washed with $NaHCO_3$aq, a 5% citric acid solution, and water, and then dried *in vacuo*. The residue was dissolved in anhydrous THF (5 mL), and cannulated into a solution of *N*-methoxycarbonylmaleimide (39.4 mg, 0.254 mmol) in anhydrous THF (10 mL). The mixture was then stirred for 5 h at room temperature. To the reaction mixture was added $NaHCO_3$aq, and the suspension was stirred for 30 min. The product was extracted with $CHCl_3$, and the organic phase was washed with H_2O and brine, and dried *in vacuo*. The residue was purified by silica gel column chromatography ($CHCl_3$/MeOH = 99/1) to afford complex **S3** as red oil. Yield: 34%. ^1H NMR (400

MHz, $CDCl_3$): δ 6.70 (s, 2H), 4.08−4.18 (m, 8H), 3.60−3.70 (m, 18H), 2.59 (t, J = 7.1 Hz, 2H), 2.44−2.51 (m, 8H), 1.77 (s, 6H), 1.74 (s, 6H), 1.45 (s, 9H), 1.43 (s, 18H), 1.42 (s, 9H). ^{13}C NMR (100 MHz, $CDCl_3$): δ 170.98, 170.89, 170.83, 170.27, 134.37, 98.83 (d, $^1J_{RhC}$ = 7.0 Hz), 98.30 (d, $^1J_{RhC}$ = 6.7 Hz), 94.53 (d, $^1J_{RhC}$ = 7.7 Hz), 80.80, 80.61, 70.21 (d, $^3J_{PC}$ = 8.5 Hz), 69.90 (d, $^3J_{PC}$ = 7.4 Hz), 69.64 (d, $^3J_{PC}$ = 9.0 Hz), 67.36 (d, $^2J_{PC}$ = 4.4 Hz), 67.07, 66.87, 65.21 (d, $^2J_{PC}$ = 8.9 Hz), 65.08 (d, $^2J_{PC}$ = 7.4 Hz), 36.44, 36.36, 35.42, 28.25, 24.01, 9.63, 9.59. ^{31}P NMR (162 MHz, $CDCl_3$): δ 117.13 (s), 97.13 (d, $^2J_{RhP}$ = 12.9 Hz). ESI-TOF MS (positive mode): *m*/*z* calcd. for $C_{33}H_{52}NO_{10}PS_2Rh$ [M – $S_2P(OC_2H_4OC_2H_4CO_2{}^tBu)_2$]$^+$ 820.183, found 820.130.

***Synthesis of 1′*:** Complex **S3** was dissolved in 2 mL of anhydrous formic acid at 0 °C and the reaction mixture was stirred at room temperature for 2 h. After removal of formic acid under reduced pressure, the residue was dissolved in AcOH, and dried *in vacuo* again to completely remove trace amounts of formic acid. ^{1}H NMR (400 MHz, acetic acid-d_4): δ 6.84 (s, 2H), 4.15−4.22 (m, 8H), 3.67−3.85 (m, 18H), 2.62−2.70 (m, 10H), 1.81 (s, 6H), 1.77 (s, 6H). ^{13}C NMR (100 MHz, acetic acid-d_4): δ 172.24, 135.44, 99.94, 99.36, 95.80, 71.09, 70.56, 67.96, 67.19, 66.32, 65.99, 36.05, 35.43, 30.60, 24.85, 9.98, 9.75. ^{31}P NMR (162 MHz, acetic acid-d_4): δ 117.49 (s), 97.62 (s). ESI-TOF MS (positive mode): *m*/*z* calcd. for $C_{25}H_{36}NO_{10}PS_2Rh$ [M – $S_2P(OC_2H_4OC_2H_4CO_2H)_2$]$^+$ 708.057, found 708.032.

***Synthesis of substrates 2b and 2c*:** A mixture of ketone (15 mmol) and hydroxylamine hydrochloride (2.50 g, 36 mmol) in a solution of EtOH (50 mL) with 5 N NaOHaq (8 mL) was refluxed overnight. After extraction with EtOAc, the organic layer was washed twice with H_2O, and dried *in vacuo*. The resulting residue was recrystallized from EtOAc to afford oximes **2b** and **2c**.

2b: ^{1}H NMR (400 MHz, $CDCl_3$): δ 7.98 (s, 1H), 7.17 (d, J = 1.7 Hz, 1H). 7.10 (dd, J = 8.2 Hz, J = 1.7 Hz, 1H), 6.80 (d, J = 8.2 Hz, 1H), 5.99 (s, 2H), 2.24 (s, 3H). ^{13}C NMR (100 MHz, $CDCl_3$): δ 155.60, 148.56, 147.91, 130.74, 120.34, 108.04, 106.21, 101.29, 12.11. ESI-TOF MS (positive mode): *m*/*z* calcd. for $C_9H_{10}NO_3$ [M + H]$^+$ 180.065, found 180.066.

2c: ^{1}H NMR (400 MHz, $CDCl_3$): δ 9.15 (s, 1H), 7.26 (s, 1H), 7.22 (d, J = 8.2 Hz, 1H), 6.96 (d, J = 8.2 Hz, 1H), 4.24 (m, 4H), 2.25 (s, 3H), 2.20 (m, 2H). ^{13}C NMR (100 MHz, $CDCl_3$): δ 155.23, 152.14, 150.97, 131.92, 121.64, 121.21, 119.57, 70.63, 70.58, 31.69, 12.28. ESI-TOF MS (positive mode): *m*/*z* calcd. for $C_{11}H_{14}NO_3$ [M + H]$^+$ 208.097, found 208.098.

***Synthesis of authentic samples 4 and 5*:** Authentic samples of **4** and **5** were synthesized according to the literature with slight modifications.[4] Oxime **2** (1.0 mmol), alkyne **3** (1.3 mmol), $[Cp^*RhCl_2]_2$ (12.5 mg, 0.02 mmol) and cesium acetate (58 mg, 0.3 mmol) were dissolved in MeOH (4 mL) and stirred for 12 h at 60 °C. After the extraction with $CHCl_3$, the organic layer was washed with $NaHCO_3$aq twice and dried *in vacuo*. The residue was purified with silica gel column chromatography (hexane/EtOAc) to obtain both regioisomers **4** and **5**.

4aa: ^{1}H NMR (400 MHz, $CDCl_3$): δ 7.63 (d, J = 9.0 Hz, 1H), 7.15 (d, J = 9.0 Hz, 1H), 5.06 (s, 2H), 4.76 (s, 2H), 4.41 (s, 4H), 3.48 (s, 3H), 3.46 (s, 3H), 2.87 (s, 3H). ^{13}C NMR (100 MHz, $CDCl_3$): δ 157.98, 150.27, 143.59, 138.63, 128.51, 124.09, 123.12, 119.75, 119.49, 74.49, 68.30, 64.34, 64.02, 58.78, 58.41, 23.16. ESI-TOF MS (positive mode): m/z calcd. for $C_{16}H_{20}NO_4$ $[M + H]^+$ 290.139, found 290.134.

4bb: ^{1}H NMR (400 MHz, $CDCl_3$): δ 7.83 (d, J = 8.8 Hz, 1H), 7.14−7.30 (m, 11H), 5.84 (s, 2H), 3.00 (s, 3H). ^{13}C NMR (100 MHz, $CDCl_3$): δ 157.92, 150.36, 147.76, 141.85, 141.01, 138.58, 131.33, 130.35, 127.63, 127.21, 126.96, 126.92, 124.94, 123.41, 122.68, 121.07, 111.01, 101.56, 23.58. ESI-TOF MS (positive mode): m/z calcd. for $C_{23}H_{18}NO_2$ $[M + H]^+$ 340.133, found 340.138.

4cb: ^{1}H NMR (400 MHz, $CDCl_3$): δ 7.85 (d, J = 8.8 Hz, 1H), 7.30 (d, J = 8.8 Hz, 1H), 7.11−7.22 (m, 11H), 4.16 (t, J = 5.2 Hz, 2H), 3.32 (t, J = 5.2 Hz, 2H), 3.00 (s, 3H), 1.96 (quin, J = 5.2 Hz, 2H). ^{13}C NMR (100 MHz, $CDCl_3$): δ 157.18, 153.03, 151.89, 146.82, 141.67, 141.59, 131.40, 130.40, 130.22, 127.48, 127.41, 126.83, 126.62, 125.74, 124.46, 123.08, 121.82, 71.22, 70.45, 31.64, 23.24. ESI-TOF MS (positive mode): m/z calcd. for $C_{25}H_{22}NO_2$ $[M + H]^+$ 368.165, found 368.171.

5cb: ^{1}H NMR (400 MHz, $CDCl_3$): δ 7.73 (s, 1H), 7.30−7.34 (m, 5H), 7.15−7.20 (m, 6H), 4.33 (t, J = 5.6 Hz, 2H), 4.28 (t, J = 5.6 Hz, 2H), 2.96 (s, 3H), 2.26 (quin, J = 5.6 Hz, 2H). ^{13}C NMR (100 MHz, $CDCl_3$): δ 156.41, 154.44, 151.26, 148.77, 141.24, 137.88, 133.82, 131.41, 130.35, 128.37, 127.69, 127.20, 126.91, 123.62, 116.45, 116.11. 70.77, 70.54, 31.35, 22.88. ESI-TOF MS (positive mode): m/z calcd. for $C_{25}H_{22}NO_2$ $[M + H]^+$ 368.165, found 368.173.

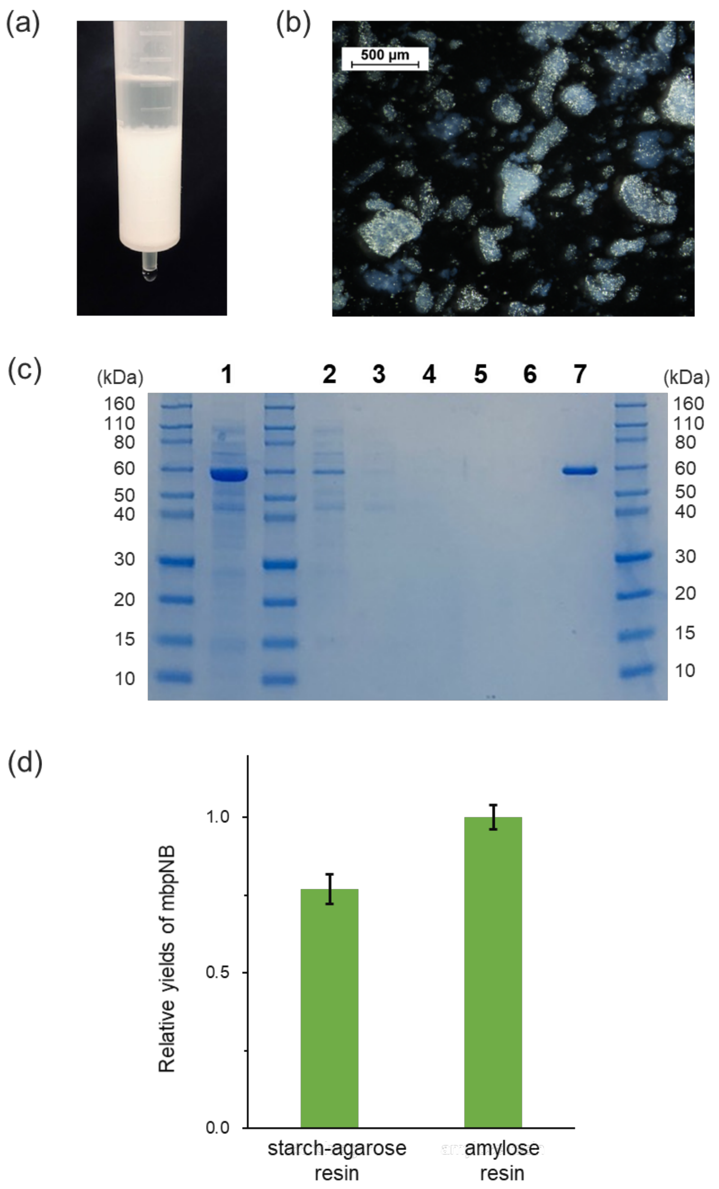

Figure S1. (a) A photograph of starch-agarose resin. (b) An optical microscope image of starch-agarose. (c) SDS-PAGE analysis for the purification of mbpNB using starch-agarose resin. mbpNB is a fusion protein of NB with the MBP-tag (mbpNB: 61 kDa). Lane 1: crude cell lysate, lanes 2–6: Eluted fractions from the starch-agarose resin washed with an AcOH-MES buffer solution, and lane 7: First fraction eluted with AcOH-MES buffer containing 25 mM maltose. (d) Relative yields of mbpNB purified using the starch-agarose resin and commercially available amylose resin (New England Biolabs Japan).

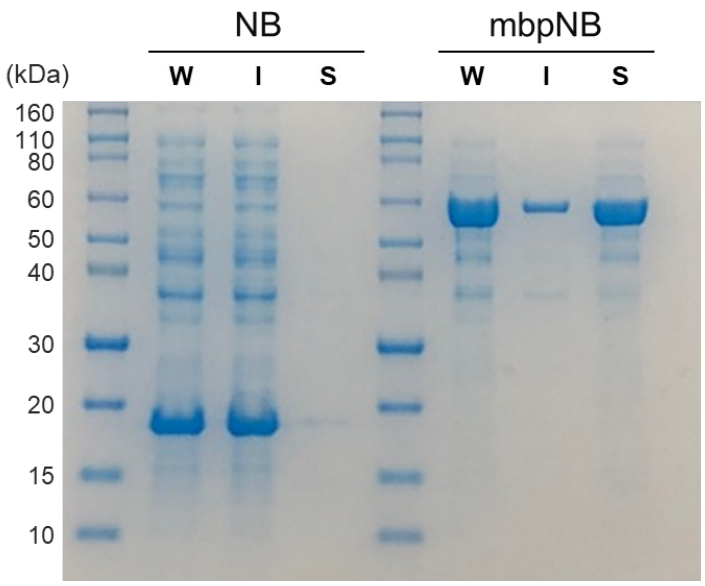

Figure S2. SDS-PAGE analysis of soluble and insoluble fractions of *E. coli* cells expressing NB or mbpNB (W: whole cell lysate, I: insoluble fraction, S: soluble fraction). mbpNB is a fusion protein of NB with the MBP-tag (NB: 19 kDa, mbpNB: 61 kDa). The fractions were prepared using the standard procedure in the HTS. *E. coli* cells were lysed with lysozyme (5.0 ng) and benzonase® nuclease (1.25 U) in 155 μL of KPi buffer (20 mM KPi, 200 mM NaCl, pH 7.0) at 37 °C for 1 h.

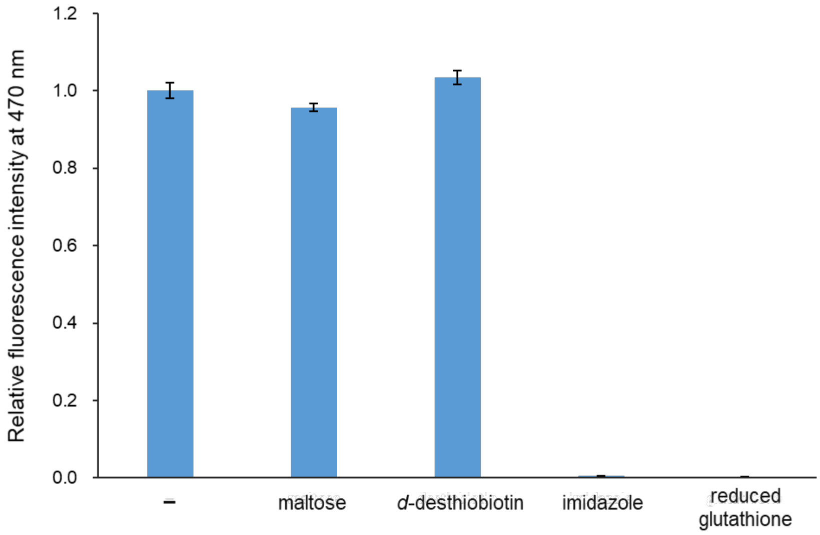

Figure S3. Cycloaddition reaction of **2a** with **3a** in the presence of maltose, *d*-desthiobiotin, imidazole, and reduced glutathione. Progress of the reaction was monitored by fluorescence intensity at 470 nm derived from product **4aa**. Maltose, *d*-desthiobiotin, imidazole, and reduced glutathione are the reagents commonly used in the elution step of the affinity chromatography for MBP-tag, strep-tag II, His-tag, and GST-tag, respectively. Reaction conditions: **2a** (0.125 mM), **3a** (2.0 mM), $Cp^*Rh[S_2P(OEt)_2]_2$ (20 μM), and $AgNO_3$ (1.0 mM) in AcOH-MES buffer (100 mM AcOH, 50 mM MES, 2% 1,4-dioxane, pH 5.5) containing maltose (25 mM).

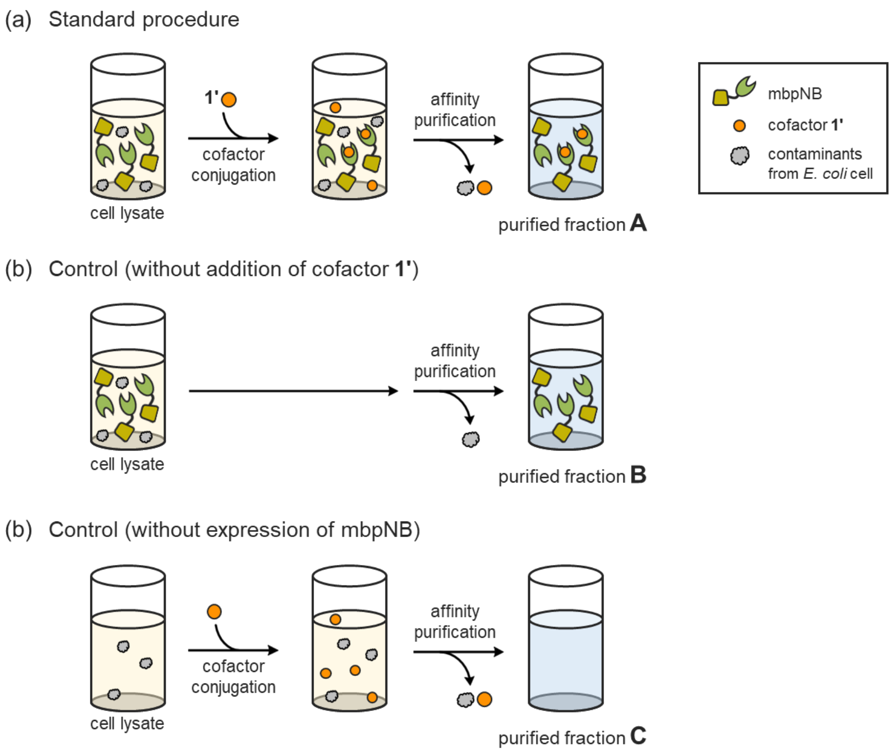

Figure S4. Procedures for preparation of the purified fractions **A**–**C** used in Figures S5–S8. (a) A standard HTS procedure for the preparation of the biohybrid catalyst mbpNB-**1′**. mbpNB in the *E. coli* cell lysate was conjugated with cofactor **1′** and the conjugate was purified using starch-agarose resin to obtain purified fraction **A**. (b) A control experiment to obtain purified fraction **B**. mbpNB in the *E. coli* cell lysate was directly purified by starch-agarose resin without the addition of cofactor **1′**. (c) A control experiment to obtain purified fraction **C**. The lysate of *E. coli* cell without IPTG induction of mbpNB expression was subjected to the process of the cofactor conjugation and the affinity purification.

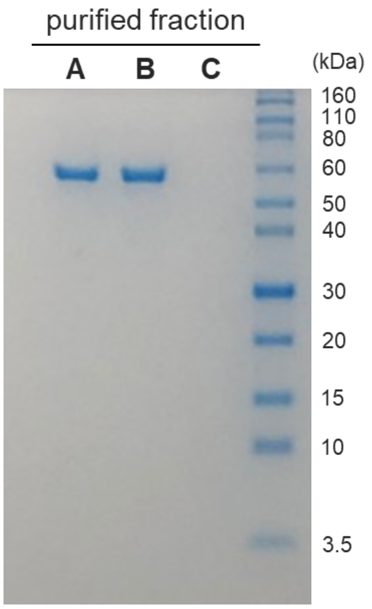

Figure S5. SDS-PAGE analysis of the purified fractions **A**–**C** prepared as shown in Figure S4. (mbpNB: 61 kDa.)

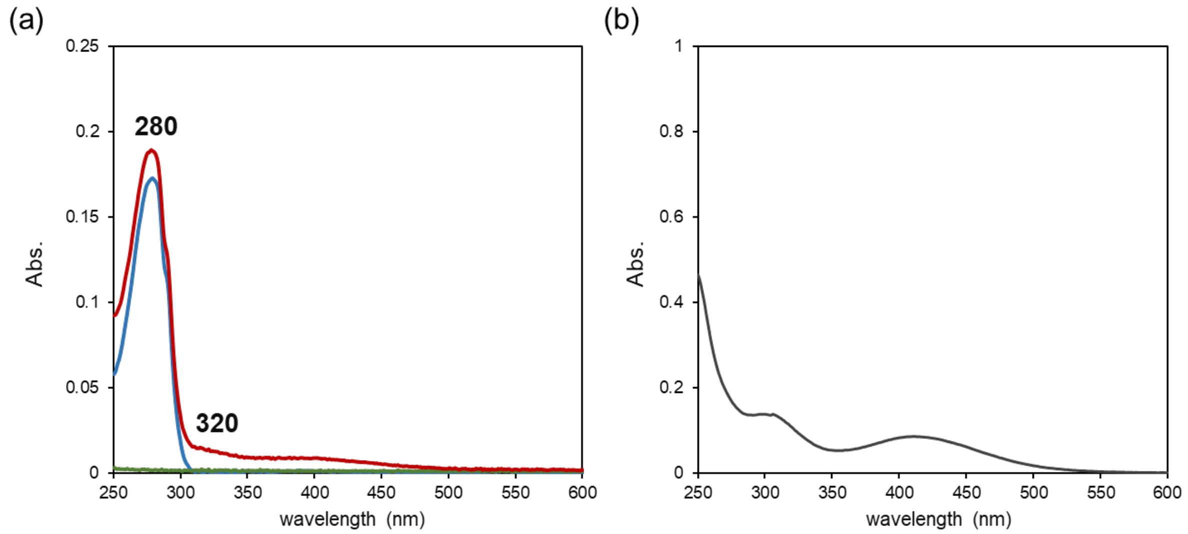

Figure S6. (a) UV-vis spectra of the purified fractions **A** (red), **B** (blue), and **C** (green), which are prepared as shown in Figure S4, in AcOH-MES buffer (50 mM MES, 100 mM AcOH, 25 mM maltose, 0.25 wt% SDS, pH 5.5) (b) UV-vis spectrum of cofactor **1′** in AcOH-MES buffer (50 mM MES, 100 mM AcOH, 25 mM maltose, pH 5.5).

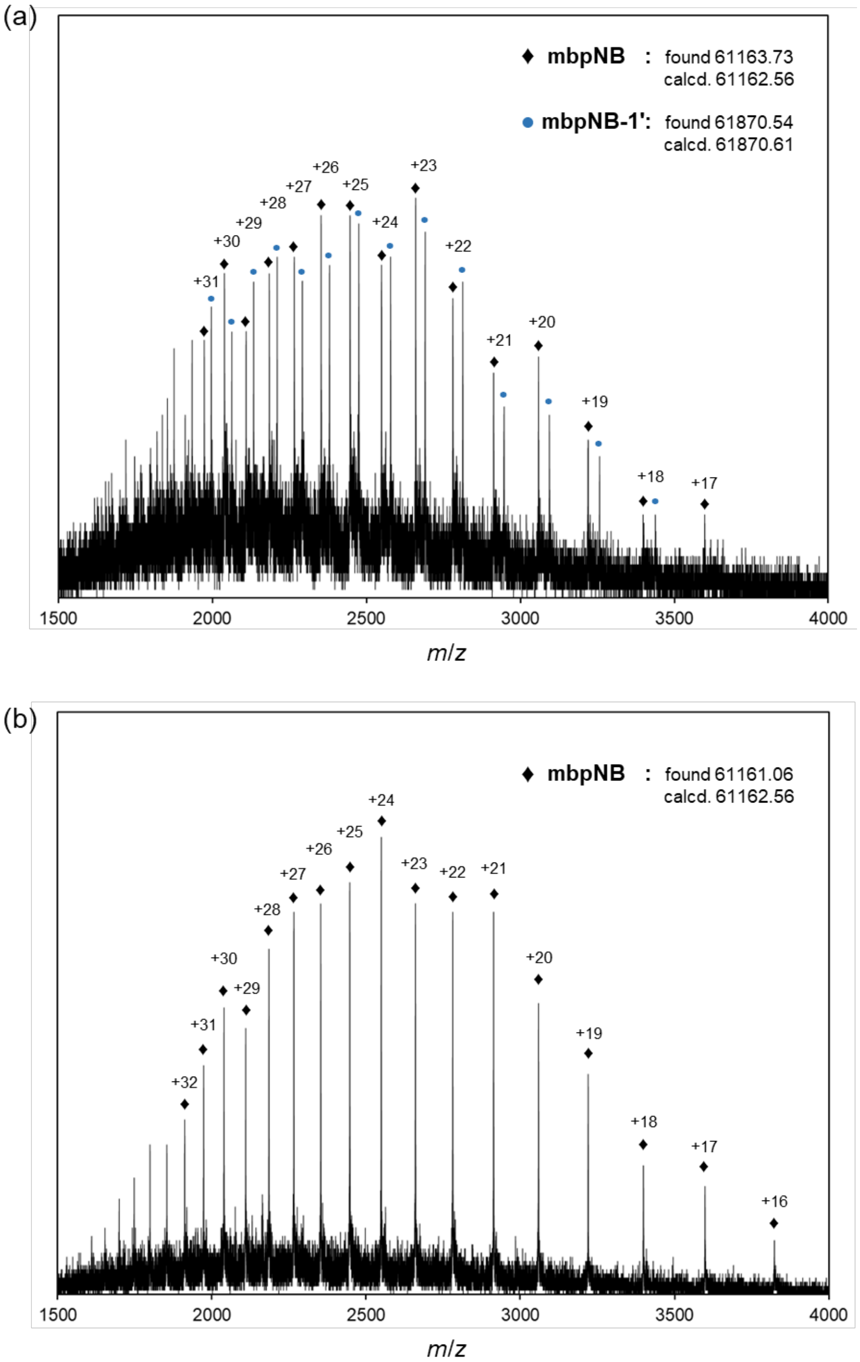

Figure S7. ESI-TOF MS of (a) purified fraction **A** and (b) purified fraction **B** prepared as shown in Figure S4.

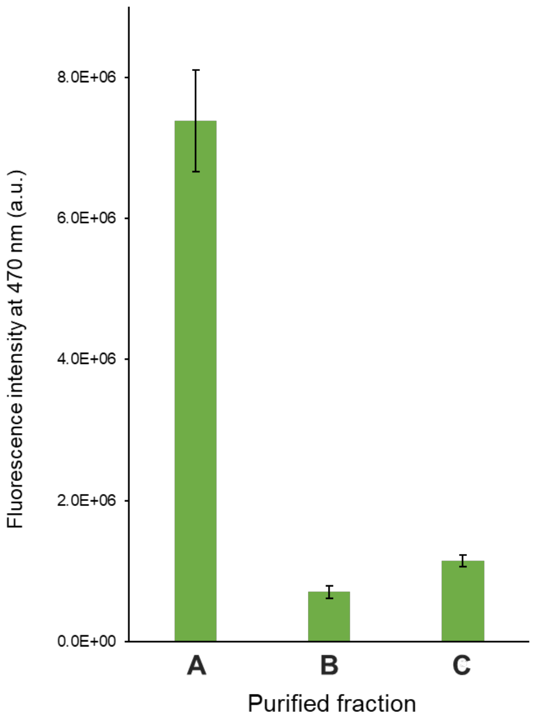

Figure S8. Cycloaddition of **2a** with **3a** using the purified fractions **A**–**C** prepared as shown in Figure S4. Reaction progress was monitored by fluorescence intensity at 470 nm derived from product **4aa**. Reaction conditions: **2a** (0.125 mM), **3a** (2.0 mM) and $AgNO_3$ (1.0 mM) in maltose elution buffer (25 mM maltose, 100 mM AcOH, 50 mM MES, 2% 1,4-dioxane, pH 5.5) containing mbpNB-**1′** (c.a. 2 μM) at 37 ºC for 72 h.

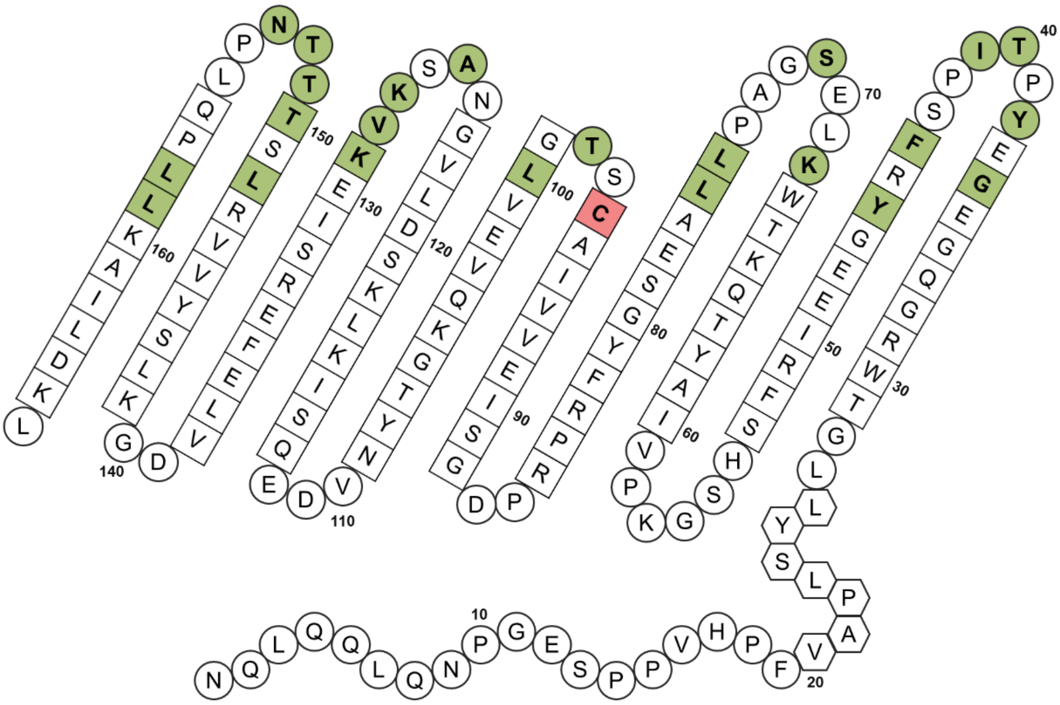

Figure S9. A topology model of NB based on the crystal structure (PDB: 3WJB). Residues in the β-strands are shown in squares and residues in the α-helix structure are shown in hexagons. Cys96 residue is highlighted in red. The twenty-three positions subjected to site-saturation mutagenesis are highlighted in green.

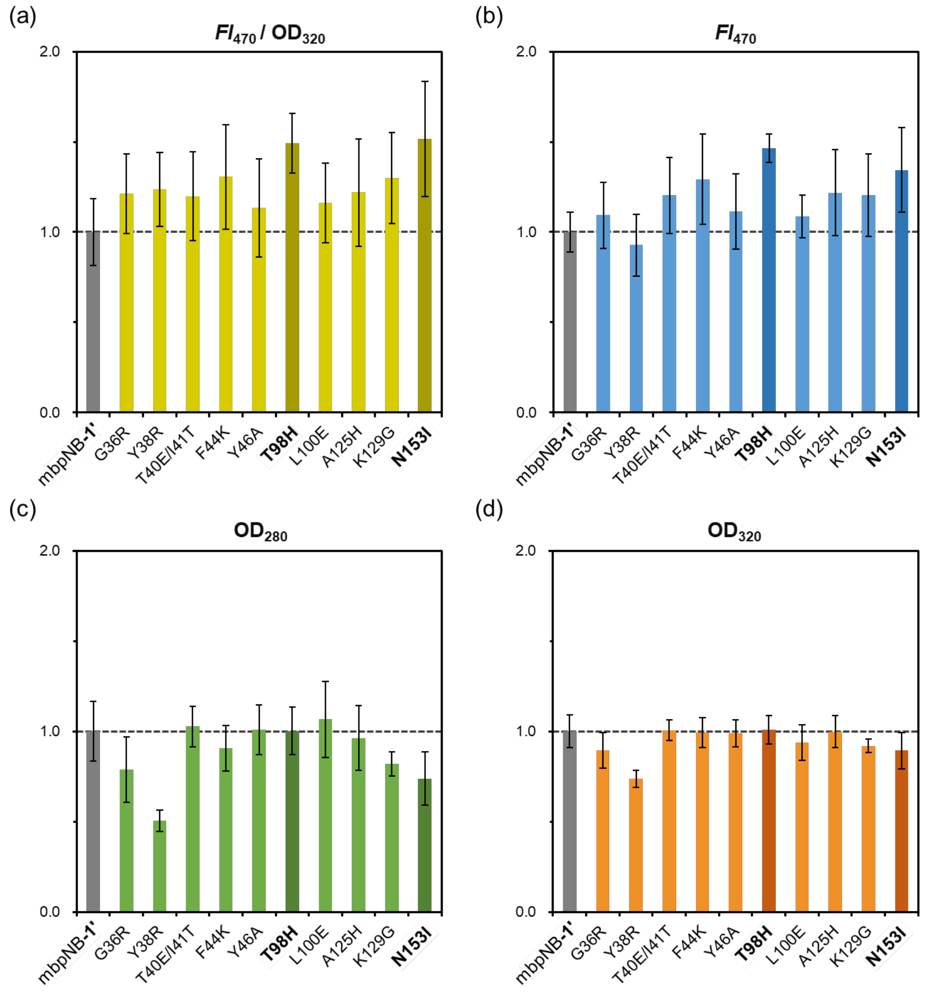

Figure S10. mbpNB-**1′** variants identified in the first round of directed evolution. (a) Relative catalytic activity of the mbpNB-**1′** variants based on FI_{470}/OD_{320}. (b) Relative fluorescence intensity at 470 nm of reaction mixture (**2a** + **3a**). (c) Relative absorbance at 280 nm of the purified fraction containing each mbpNB-**1′** variant. (d) Relative absorbance at 320 nm of the purified fraction containing each mbpNB-**1′** variant.

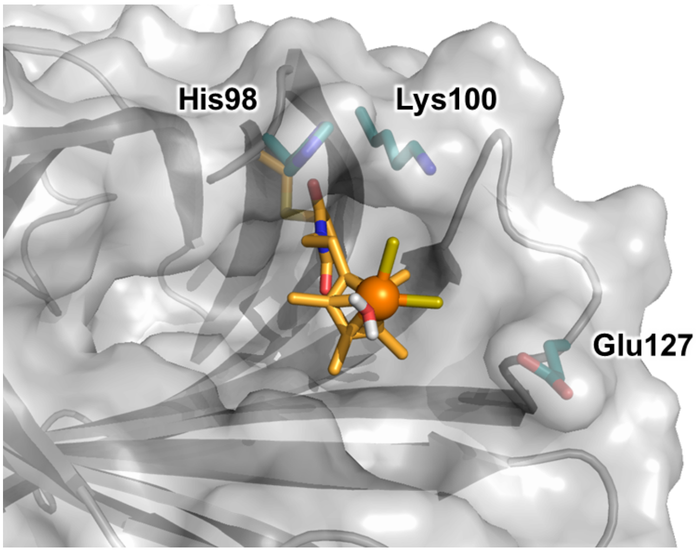

Figure S11. An MD structure of Cp*Rh(III)-linked NB(T98H/L100K/K127E). The MD calculation was carried out based on the crystal structure of NB (PDB: 3WJB). Two dithiophosphate ligands of cofactor **1** were substituted with two chloride ions and one water molecule. His98, Lys100 and Glu127 are highlighted as green sticks.

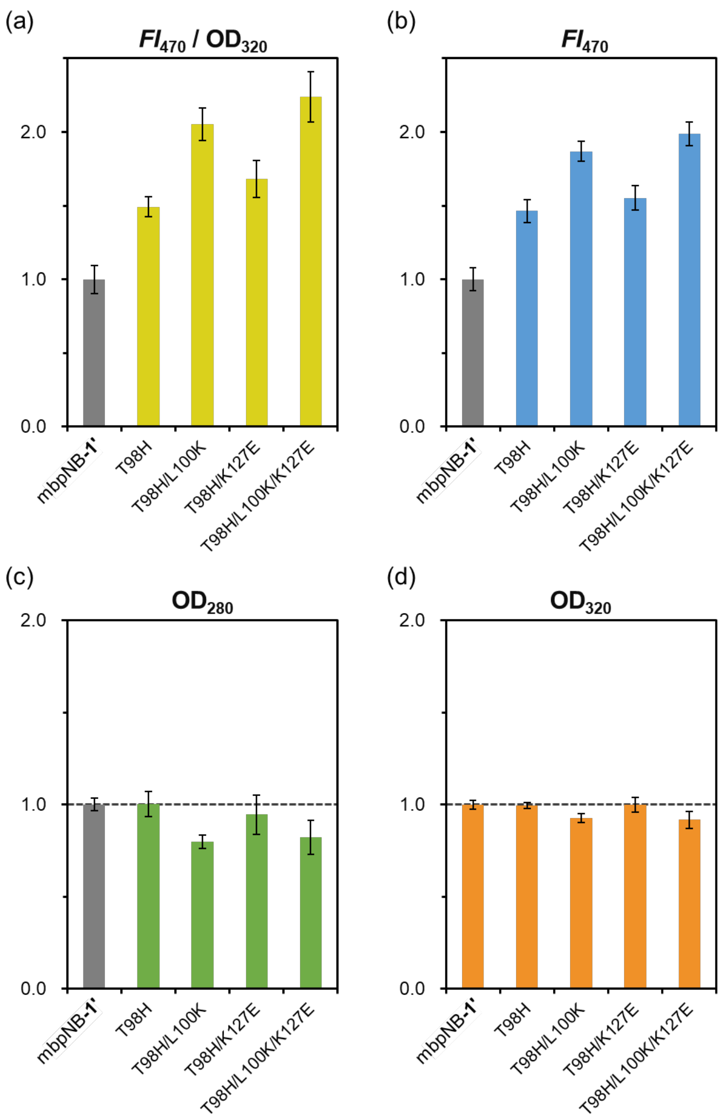

Figure S12. mbpNB-**1**′ variants identified through the three rounds of directed evolution. (a) Relative catalytic activity of the mbpNB-**1**′ variants based on FI_{470}/OD_{320}. (b) Relative fluorescence intensity at 470 nm (FI_{470}) of reaction mixture (**2a** + **3a**). (c) Relative absorbance at 280 nm (OD_{280}) of the purified fraction containing each mbpNB-**1**′ variant. (d) Relative absorbance at 320 nm (OD_{320}) of the purified fraction containing each mbpNB-**1**′ variant.

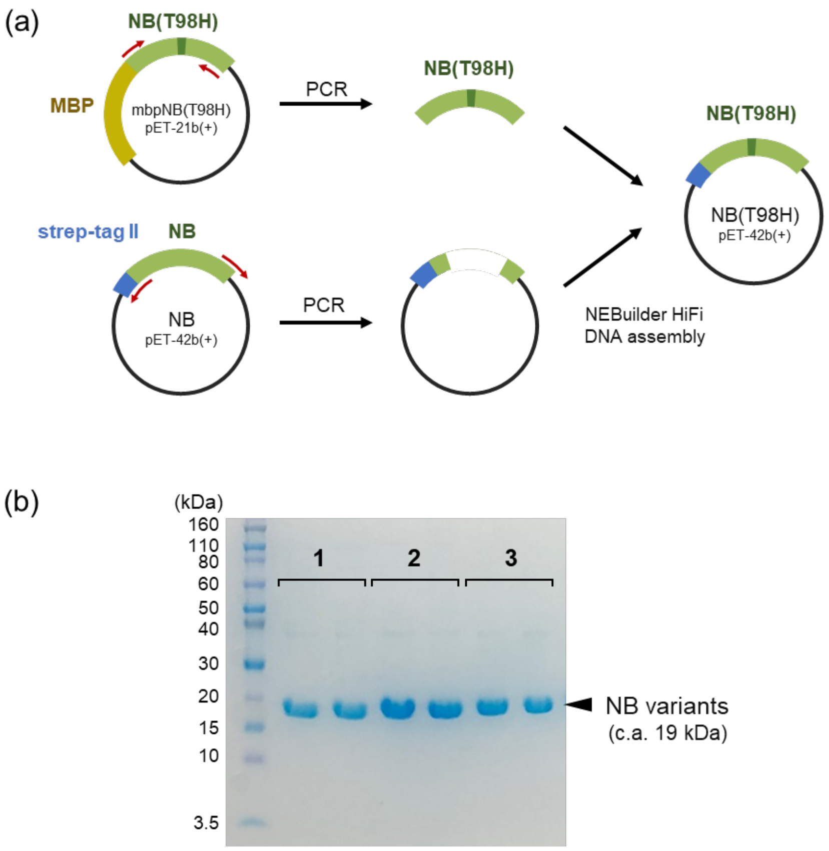

Figure S13. (a) Schematic illustration of the subcloning of an NB(T98H) gene into pET42b(+) vector with a strep-tag II gene based on NEBuilder HiFi DNA assembly technique. (b) SDS-PAGE analysis of the purified NB variants with strep-tag II. Lanes 1: NB(T98H), lanes 2: NB(T98H/L100K), lanes 3: NB(T98H/L100K/K127E).

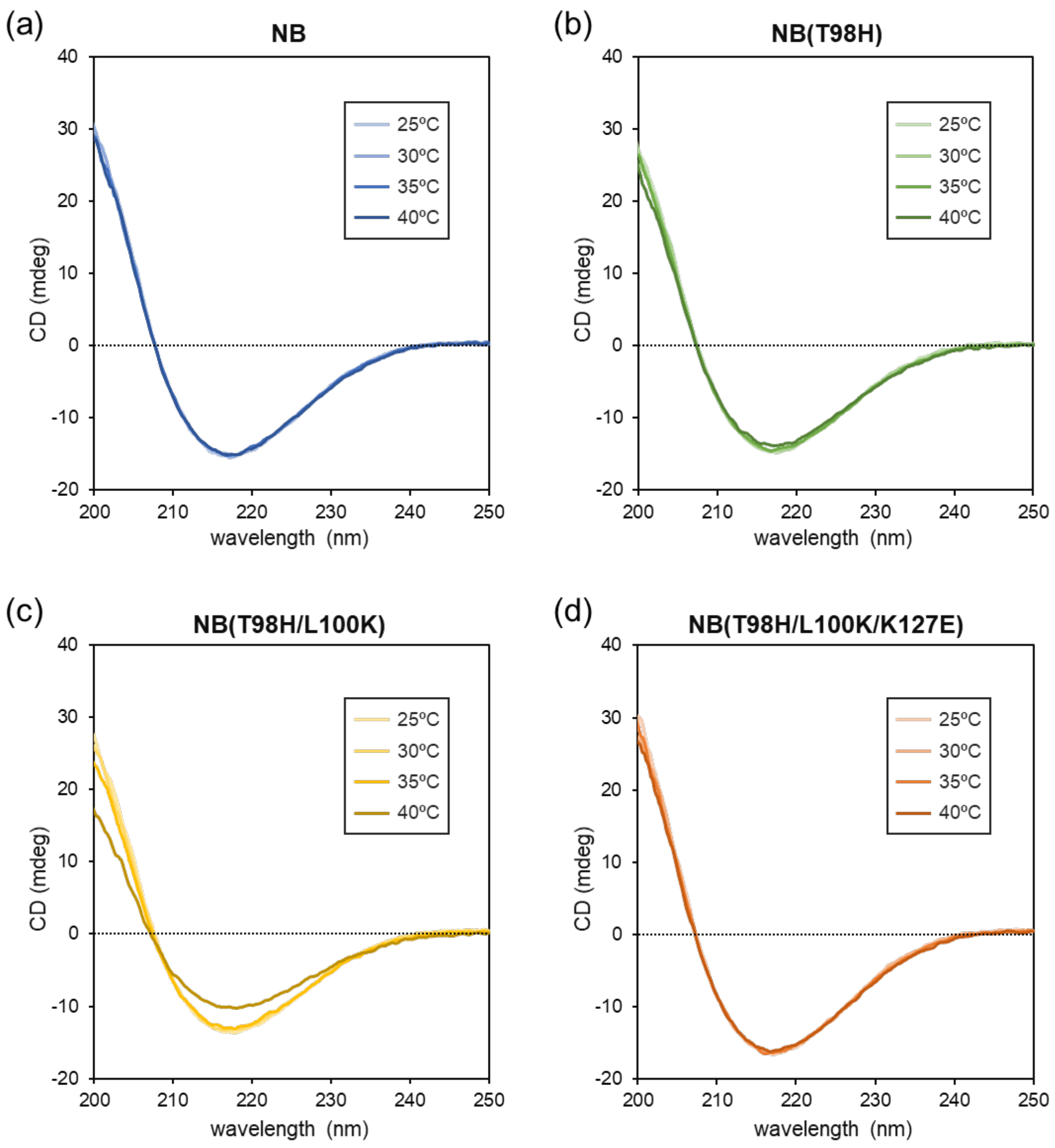

Figure S14. CD spectra of the purified NB variants in KPi buffer (10 mM KPi, pH 7.0). (a) NB, (b) NB(T98H), (c) NB(T98H/L100K), and (d) NB(T98H/L100K/K127E).

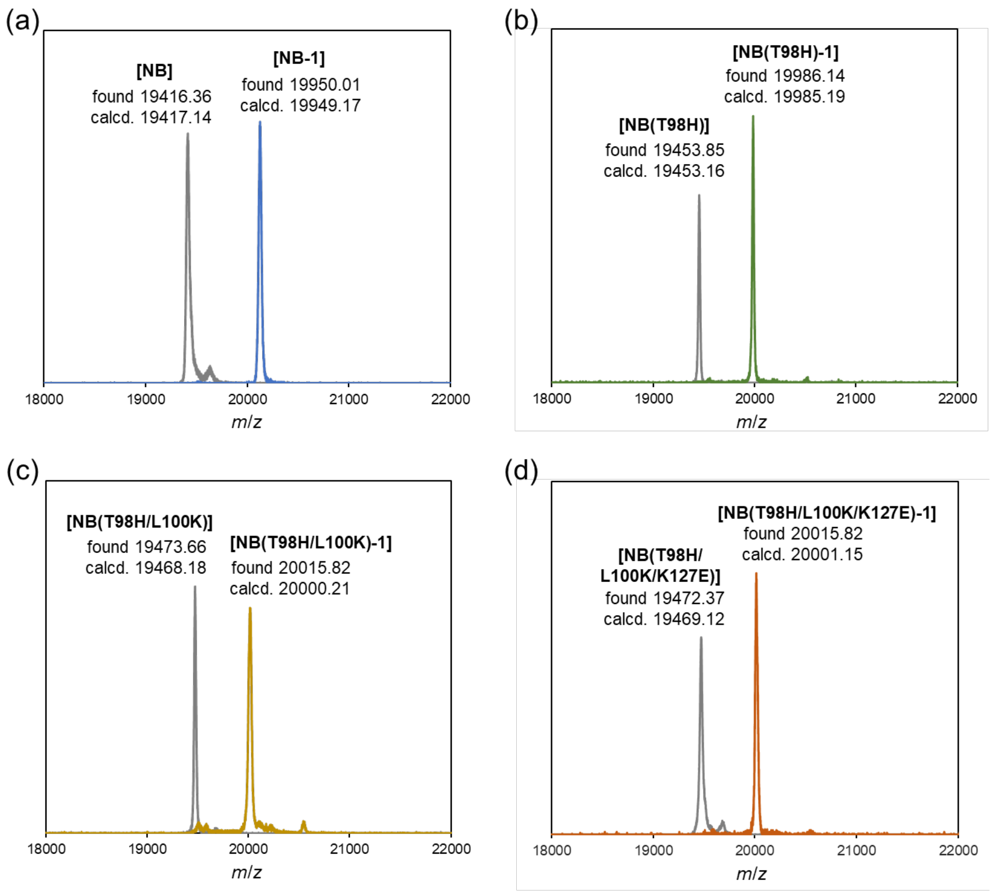

Figure S15. MALDI-TOF MS of (a) NB (gray) and NB-**1** (blue), (b) NB(T98H) (gray) and NB(T98H)-**1** (green), (c) NB(T98H/L100K) (gray) and NB(T98H/L100K)-**1** (yellow), and (d) NB(T98H/L100K/K127E) (gray) and NB(T98H/L100K/K127E)-**1** (orange).

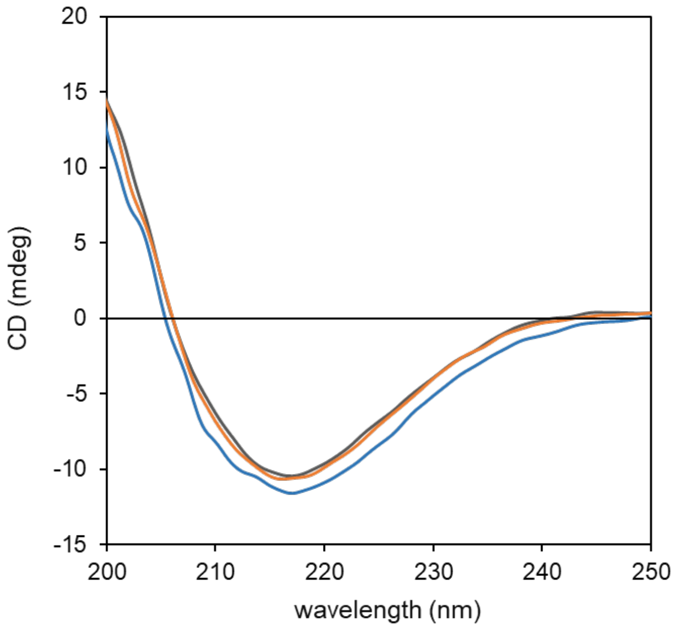

Figure S16. CD spectra of NB-**1** in AcOH buffer (gray), NB-**1** in AcOH buffer containing 2.0 v/v% of 1,4-dioxane (orange), and NB-**1** in AcOH buffer containing 20 v/v% of THF (blue).

Table S1. Preparation Cost for 50 mL of Starch-Agarose Resin[a]

Chemicals	Price (JPY)	Required Amounts	Cost (JPY)
agarose	86,500 (500 g)	1.6 g	276.8
soluble starch	1,750 (500 g)	0.6 g	2.1
NaCl	2,000 (500 g)	0.36 g	1.4
paraffin oil	7,040 (500 mL)	70 mL	985.6
Tween 80	2,700 (500 mL)	2.1 mL	11.3

Total: 1,277 (JPY)

[a]In the high-throughput screening for a biohybrid catalyst, c.a. 50 mL starch-agarose resin was packed into one 96-well filter plate (0.50 mL per each well).

Table S2. Degenerate Codons and Amino Acids Encoded

entry	Codons[a]	Amino acids encoded
1	NHN	ANDQEHILKMFPSTYV
2	VNN	ARNDQEGHILKMPSTV
3	TGG	W

(N = A/C/G/T, H = A/C/T, V = A/C/G, K = G/T)

[a] Three oligonucleotide primers containing NHN, VNN, and TGG codons were mixed in a ratio of 10:10:1 and used for site-saturation mutagenesis.

Table S3. Cycloaddition of 2a with 3a Catalyzed by NB-1 Variants[a]

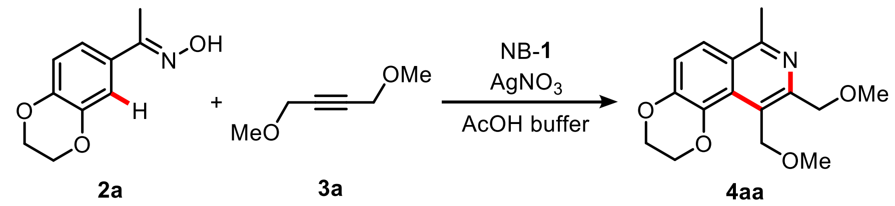

entry	Catalyst	Yield (%)[b]
1	NB-**1**	< 1.0
2	NB(T98H)-**1**	3.4 ± 0.1
3	NB(T98H/L100K)-**1**	3.6 ± 0.2
4	NB(T98H/L100K/K127E)-**1**	4.2 ± 0.1

[a]Reaction conditions: NB-**1** variants (20 μM), **2a** (0.125 mM), **3a** (2.0 mM) and $AgNO_3$ (0.1 mM) in AcOH buffer (100 mM AcOH, 20% THF, pH 4.0), 25ºC, 48 h. [b]Yields of product **4aa** were determined by GC-MS.

DNA and Amino Acid Sequence

NB:

ATGTGGAGCCACCCGCAGTTCGAAAAAAATCAACTGCAACAACTGCAAAATCCGGGCG
AGAGTCCGCCGGTTCATCCGTTCGTGGCACCGCTGTCCTATCTGCTGGGTACCTGGCGCG
GCCAGGGTGAAGGCGAGTATCCGACCATTCCGAGCTTTCGCTATGGCGAAGAGATCCGT
TTCAGCCATTCGGGTAAACCGGTGATTGCCTATACCCAAAAAACGTGGAAACTGGAATC
GGGTGCACCGCTGCTGGCAGAGAGTGGTTATTTTCGCCCGCGTCCGGATGGTTCTATTG
AAGTGGTTATCGCATGCTCGACCGGTCTGGTGGAAGTTCAAAAAGGCACGTATAATGTG
GATGAGCAGAGTATTAAACTGAAATCTGACCTGGTGGGCAACGCGTCCAAAGTTAAAG
AAATCAGCCGCGAATTCGAGCTGGTTGACGGTAAACTGAGTTATGTGGTTCGTCTGAGC
ACGACCACGAATCCGCTGCAACCGCTGCTGAAAGCCATCCTGGACAAACTG

(blue: Strep-tag II, black: NB)

MWSHPQFEKNQLQQLQNPGESPPVHPFVAPLSYLLGTWRGQGEGEYPTIPSFRYGEEIRFSH
SGKPVIAYTQKTWKLESGAPLLAESGYFRPRPDGSIEVVIACSTGLVEVQKGTYNVDEQSIKL
KSDLVGNASKVKEISREFELVDGKLSYVVRLSTTTNPLQPLLKAILDKL

(blue: Strep-tag II, black: NB)

mbpNB:

ATGAAAATCGAAGAAGGTAAACTGGTAATCTGGATTAACGGCGATAAAGGCTATAACG
GTCTCGCTGAAGTCGGTAAGAAATTCGAGAAAGATACCGGAATTAAAGTCACCGTTGAG
CATCCGGATAAACTGGAAGAGAAATTCCCACAGGTTGCGGCAACTGGCGATGGCCCTG
ACATTATCTTCTGGGCACACGACCGCTTTGGTGGCTACGCTCAATCTGGCCTGTTGGCTG
AAATCACCCCGGACAAAGCGTTCCAGGACAAGCTGTATCCGTTTACCTGGGATGCCGTA
CGTTACAACGGCAAGCTGATTGCTTACCCGATCGCTGTTGAAGCGTTATCGCTGATTTAT
AACAAAGATCTGCTGCCGAACCCGCCAAAAACCTGGGAAGAGATCCCGGCGCTGGATA
AAGAACTGAAAGCGAAAGGTAAGAGCGCGCTGATGTTCAACCTGCAAGAACCGTACTT
CACCTGGCCGCTGATTGCTGCTGACGGGGGTTATGCGTTCAAGTATGAAAACGGCAAGT
ACGACATTAAAGACGTGGGCGTGGATAACGCTGGCGCGAAAGCGGGTCTGACCTTCCTG
GTTGACCTGATTAAAAACAAACACATGAATGCAGACACCGATTACTCCATCGCAGAAGC
TGCCTTTAATAAAGGCGAAACAGCGATGACCATCAACGGCCCGTGGGCATGGTCCAACA

TCGACACCAGCAAAGTGAATTATGGTGTAACGGTACTGCCGACCTTCAAGGGTCAACCA
TCCAAACCGTTCGTTGGCGTGCTGAGCGCAGGTATTAACGCCGCCAGTCCGAACAAAGA
GCTGGCAAAAGAGTTCCTCGAAAACTATCTGCTGACTGATGAAGGTCTGGAAGCGGTTA
ATAAAGACAAACCGCTGGGTGCCGTAGCGCTGAAGTCTTACGAGGAAGAGTTGGCGAA
AGATCCACGTATTGCCGCCACCATGGAAAACGCCCAGAAAGGTGAAATCATGCCGAAC
ATCCCGCAGATGTCCGCTTTCTGGTATGCCGTGCGTACTGCGGTGATCAACGCCGCCAG
CGGTCGTCAGACTGTCGATGAAGCCCTGAAAGACGCGCAGACTGCAGAAGCAGCAGCA
AAAGAAGCCGCTGCCAAAGAAGCGGCAGCGAAAGCAAATCAACTGCAACAACTGCAAA
ATCCGGGCGAGAGTCCGCCGGTTCATCCGTTCGTGGCACCGCTGTCCTATCTGCTGGGTA
CCTGGCGCGGCCAGGGTGAAGGCGAGTATCCGACCATTCCGAGCTTTCGCTATGGCGAA
GAGATCCGTTTCAGCCATTCGGGTAAACCGGTGATTGCCTATACCCAAAAAACGTGGAA
ACTGGAATCGGGTGCACCGCTGCTGGCAGAGAGTGGTTATTTTCGCCCGCGTCCGGATG
GTTCTATTGAAGTGGTTATCGCATGCTCGACCGGTCTGGTGGAAGTTCAAAAAGGCACG
TATAATGTGGATGAGCAGAGTATTAAACTGAAATCTGACCTGGTGGGCAACGCGTCCAA
AGTTAAAGAAATCAGCCGCGAATTCGAGCTGGTTGACGGTAAACTGAGTTATGTGGTTC
GTCTGAGCACGACCACGAATCCGCTGCAACCGCTGCTGGAAGCCATCCTGGACAAACTG
TGGAGCCACCCGCAGTTCGAAAAA

(yellow: MBP-tag, green: α-helix linker, blue: Strep-tag II, black: NB)

MKIEEGKLVIWINGDKGYNGLAEVGKKFEKDTGIKVTVEHPDKLEEKFPQVAATGDGPDIIF
WAHDRFGGYAQSGLLAEITPDKAFQDKLYPFTWDAVRYNGKLIAYPIAVEALSLIYNKDLL
PNPPKTWEEIPALDKELKAKGKSALMFNLQEPYFTWPLIAADGGYAFKYENGKYDIKDVGV
DNAGAKAGLTFLVDLIKNKHMNADTDYSIAEAAFNKGETAMTINGPWAWSNIDTSKVNYG
VTVLPTFKGQPSKPFVGVLSAGINAASPNKELAKEFLENYLLTDEGLEAVNKDKPLGAVAL
KSYEEELAKDPRIAATMENAQKGEIMPNIPQMSAFWYAVRTAVINAASGRQTVDEALKDA
QTAEAAAKEAAAKEAAAKANQLQQLQNPGESPPVHPFVAPLSYLLGTWRGQGEGEYPTIPS
FRYGEEIRFSHSGKPVIAYTQKTWKLESGAPLLAESGYFRPRPDGSIEVVIACSTGLVEVQKG
TYNVDEQSIKLKSDLVGNASKVKEISREFELVDGKLSYVVRLSTTTNPLQPLLEAILDKLWS
HPQFEK

(yellow: MBP-tag, green: α-helix linker, blue: Strep-tag II, black: NB)

Primers for construction of expression plasmid for fusion protein of mbpNB

pET-21b(+) with NB vector, forward primer

5'-GGTAGTGAAAACCTGTACTTCCAGGGTAATCAACTGCAACAACTGCAAAATCC-3'

pET-21b(+) with NB vector, reverse primer

5'-CATATGTATATCTCCTTCTTAAAGTTAAACAAAATTATTTCTAGAGG-3'

MBP insert, forward primer

5'-GGAGATATACATATGAAAATCGAAGAAGGTAAACTGGTAATCTG-3'

MBP insert, reverse primer

5'-CAGGTTTTCACTACCACTACCACTACCAGTCTGCGCGTCTTTCAGGG-3'

pET-21b(+) vector with NB and MBP, forward primer

5'-AATCAACTGCAACAACTGC-3'

pET-21b(+) vector with NB and MBP, reverse primer

5'-AGTCTGCGCGTCTTTCAG-3'

α-helix linker insert, forward primer

5'-CTGAAAGACGCGCAGACTGCAGAAGCAGCAGCAAAAG-3'

α-helix linker insert, reverse primer

5'-GCAGTTGTTGCAGTTGATTTGCTTTCGCTGCCGCTTC-3'

Forward and reverse primers for site-saturation mutagenesis

G36, forward primer

5'-GCCAGGGTGAA**NHN**GAGTATCCGACCATTC-3'

5'-GCCAGGGTGAA**VNN**GAGTATCCGACCATTC-3'

5'-GCCAGGGTGAA**TGG**GAGTATCCGACCATTC-3'

G36, reverse primer

5'-GAATGGTCGGATACTC**NDN**TTCACCCTGGC-3'

5'-GAATGGTCGGATACTC**NNB**TTCACCCTGGC-3'

5’-GAATGGTCGGATACTC**CAA**TTCACCCTGGC-3’

Y38, forward primer

5’-CAGGGTGAAGGCGAG**NHN**CCGACCATTCCGAGC-3’

5’-CAGGGTGAAGGCGAG**VNN**CCGACCATTCCGAGC-3’

5’-CAGGGTGAAGGCGAG**TGG**CCGACCATTCCGAGC-3’

Y38, reverse primer

5’-GCTCGGAATGGTCGG**NDN**CTCGCCTTCACCCTG-3’

5’-GCTCGGAATGGTCGG**NNB**CTCGCCTTCACCCTG-3’

5’-GCTCGGAATGGTCGG**CAA**CTCGCCTTCACCCTG-3’

T40/I41, forward primer

5’-CAGGGTGAAGGCGAGTATCCG**NHNNHN**CCGAGCTTTCGCTATGG-3’

T40/I41, reverse primer

5’-CCATAGCGAAAGCTCGG**NDNNDN**CGGATACTCGCCTTCACCCTG-3’

F44, forward primer

5’-GACCATTCCGAGC**NHN**CGCTATGGCGAAGAGATC-3’

5’-GACCATTCCGAGC**VNN**CGCTATGGCGAAGAGATC-3’

5’-GACCATTCCGAGC**TGG**CGCTATGGCGAAGAGATC-3’

F44, reverse primer

5’-GATCTCTTCGCCATAGCG**NDN**GCTCGGAATGGTC-3’

5’-GATCTCTTCGCCATAGCG**NNB**GCTCGGAATGGTC-3’

5’-GATCTCTTCGCCATAGCG**CCA**GCTCGGAATGGTC-3’

Y46, forward primer

5’-CCATTCCGAGCTTTCGC**NHN**GGCGAAGAGATC-3’

5’-CCATTCCGAGCTTTCGC**VNN**GGCGAAGAGATC-3’

5’-CCATTCCGAGCTTTCGC**TGG**GGCGAAGAGATC-3’

Y46, reverse primer

5’-GATCTCTTCGCC**NDN**GCGAAAGCTCGGAATGG-3’

5’-GATCTCTTCGCC**NNB**GCGAAAGCTCGGAATGG-3’

5’-GATCTCTTCGCC**CCA**GCGAAAGCTCGGAATGG-3’

K68/S71, forward primer

5'-CCTATACCCAAAAAACGTGG**NHN**CTGGAA**NHN**GGTGCACCGCTG-3'

K68/S71, reverse primer

5'-CAGCGGTGCACC**NDN**TTCCAG**NDN**CCACGTTTTTTGGGTATAGG-3'

S71, forward primer

5'-CGTGGAAACTGGAA**NHN**GGTGCACCGCTG-3'

5'-CGTGGAAACTGGAA**VNN**GGTGCACCGCTG-3'

5'-CGTGGAAACTGGAA**TGG**GGTGCACCGCTG-3'

S71, reverse primer

5'-CAGCGGTGCACC**NDN**TTCCAGTTTCCACG-3'

5'-CAGCGGTGCACC**NNB**TTCCAGTTTCCACG-3'

5'-CAGCGGTGCACC**CCA**TTCCAGTTTCCACG-3'

L75/L76, forward primer

5'-CTGGAATCGGGTGCACCG**NHNNHN**GCAGAGAGTGGTTATTTTCGC-3'

L75/L76, reverse primer

5'-GCGAAAATAACCACTCTCTGC**NDNNDN**CGGTGCACCCGATTCCAG-3'

T98, forward primer

5'-GGTTATCGCATGCTCG**NHN**GGTCTGGTGGAAG-3'

5'-GGTTATCGCATGCTCG**VNN**GGTCTGGTGGAAG-3'

5'-GGTTATCGCATGCTCG**TGG**GGTCTGGTGGAAG-3'

T98, reverse primer

5'-CTTCCACCAGACC**NDN**CGAGCATGCGATAACC-3'

5'-CTTCCACCAGACC**NNB**CGAGCATGCGATAACC-3'

5'-CTTCCACCAGACC**CCA**CGAGCATGCGATAACC-3'

L100, forward primer

5'-GCATGCTCGACCGGT**NHN**GTGGAAGTTC-3'

5'-GCATGCTCGACCGGT**VNN**GTGGAAGTTC-3'

5'-GCATGCTCGACCGGT**TGG**GTGGAAGTTC-3'

L100, reverse primer

5'-GAACTTCCAC**NDN**ACCGGTCGAGCATGC-3'

5'-GAACTTCCAC**NNB**ACCGGTCGAGCATGC-3'

5'-GAACTTCCAC**CCA**ACCGGTCGAGCATGC-3'

L100 (T98H), forward primer

5'-GCATGCTCGCACGGT**NHN**GTGGAAGTTC-3'

5'-GCATGCTCGCACGGT**VNN**GTGGAAGTTC-3'

5'-GCATGCTCGCACGGT**TGG**GTGGAAGTTC-3'

L100 (T98H), reverse primer

5'-GAACTTCCAC**NDN**ACCGTGCGAGCATGC-3'

5'-GAACTTCCAC**NNB**ACCGTGCGAGCATGC-3'

5'-GAACTTCCAC**CCA**ACCGTGCGAGCATGC-3'

A125, forward primer

5'-GACCTGGTGGGCAAC**NHN**TCCAAAGTTAAAG-3'

5'-GACCTGGTGGGCAAC**VNN**TCCAAAGTTAAAG-3'

5'-GACCTGGTGGGCAAC**TGG**TCCAAAGTTAAAG-3'

A125, reverse primer

5'-CTTTAACTTTGGA**NDN**GTTGCCCACCAGGTC-3'

5'-CTTTAACTTTGGA**NNB**GTTGCCCACCAGGTC-3'

5'-CTTTAACTTTGGA**CCA**GTTGCCCACCAGGTC-3'

K127, forward primer

5'-GTGGGCAACGCGTCC**NHN**GTTAAAGAAATCAGC-3'

5'-GTGGGCAACGCGTCC**VNN**GTTAAAGAAATCAGC-3'

5'-GTGGGCAACGCGTCC**TGG**GTTAAAGAAATCAGC-3'

K127, reverse primer

5'-GCTGATTTCTTTAAC**NDN**GGACGCGTTGCCCAC-3'

5'-GCTGATTTCTTTAAC**NNB**GGACGCGTTGCCCAC-3'

5'-GCTGATTTCTTTAAC**CCA**GGACGCGTTGCCCAC-3'

V128, forward primer

5’-GCAACGCGTCCAAA**NHN**AAAGAAATCAGCCGCGAATTC-3’

5’-GCAACGCGTCCAAA**VNN**AAAGAAATCAGCCGCGAATTC-3’

5’-GCAACGCGTCCAAA**TGG**AAAGAAATCAGCCGCGAATTC-3’

V128, reverse primer

5’-GAATTCGCGGCTGATTTCTTT**NDN**TTTGGACGCGTTGC-3’

5’-GAATTCGCGGCTGATTTCTTT**NNB**TTTGGACGCGTTGC-3’

5’-GAATTCGCGGCTGATTTCTTT**CCA**TTTGGACGCGTTGC-3’

K129, forward primer

5’-CAACGCGTCCAAAGTT**NHN**GAAATCAGCCGCGAATTCG-3’

5’-CAACGCGTCCAAAGTT**VNN**GAAATCAGCCGCGAATTCG-3’

5’-CAACGCGTCCAAAGTT**TGG**GAAATCAGCCGCGAATTCG-3’

K129, reverse primer

5’-CGAATTCGCGGCTGATTTC**NDN**AACTTTGGACGCGTTG-3’

5’-CGAATTCGCGGCTGATTTC**NNB**AACTTTGGACGCGTTG -3’

5’-CGAATTCGCGGCTGATTTC**CCA**AACTTTGGACGCGTTG -3’

L148, forward primer

5’-GAGTTATGTGGTTCGT**NHN**AGCACGACCACGAATC-3’

5’-GAGTTATGTGGTTCGT**VNN**AGCACGACCACGAATC-3’

5’-GAGTTATGTGGTTCGT**TGG**AGCACGACCACGAATC-3’

L148, reverse primer

5’-GATTCGTGGTCGTGCT**NDN**ACGAACCACATAACTC-3’

5’-GATTCGTGGTCGTGCT**NNB**ACGAACCACATAACTC-3’

5’-GATTCGTGGTCGTGCT**CCA**ACGAACCACATAACTC-3’

T150/T151/T152, forward primer

5’-GTTATGTGGTTCGTCTGAGC**NHNNHNNHN**AATCCGCTGCAACCGC-3’

T150/T151/T152, reverse primer

5’- GCGGTTGCAGCGGATT**NDNNDNNDN**GCTCAGACGAACCACATAAC-3’

N153, forward primer

5’-CTGAGCACGACCACG**NHN**CCGCTGCAACCG-3’

5’-CTGAGCACGACCACG**VNN**CCGCTGCAACCG-3’

5’-CTGAGCACGACCACG**TGG**CCGCTGCAACCG-3’

N153, reverse primer

5’-CGGTTGCAGCGG**NDN**CGTGGTCGTGCTCAG-3’

5’-CGGTTGCAGCGG**NNB**CGTGGTCGTGCTCAG-3’

5’-CGGTTGCAGCGG**CCA**CGTGGTCGTGCTCAG-3’

L158, forward primer

5’-GAATCCGCTGCAACCG**NHN**CTGGAAGCCATCC-3’

5’-GAATCCGCTGCAACCG**VNN**CTGGAAGCCATCC-3’

5’-GAATCCGCTGCAACCG**TGG**CTGGAAGCCATCC-3’

L158, reverse primer

5’-GGATGGCTTCCAG**NDN**CGGTTGCAGCGGATTC-3’

5’-GGATGGCTTCCAG**NNB**CGGTTGCAGCGGATTC-3’

5’-GGATGGCTTCCAG**CCA**CGGTTGCAGCGGATTC-3’

L159, forward primer

5’-GCAACCGCTG**NHN**GAAGCCATCCTGGAC-3’

5’-GCAACCGCTG**VNN**GAAGCCATCCTGGAC -3’

5’-GCAACCGCTG**TGG**GAAGCCATCCTGGAC -3’

L159, reverse primer

5’-GTCCAGGATGGCTTC**NDN**CAGCGGTTGC-3’

5’-GTCCAGGATGGCTTC**NNB**CAGCGGTTGC-3’

5’-GTCCAGGATGGCTTC**CCA**CAGCGGTTGC-3’

Forward and reverse primers for subcloning of evolved NB gene

Insert, forward primer

5'-CAACTGCAAAATCCGGGCGAGAGTCC-3'

Insert, reverse primer

5'-GATGGCTTTCAGCAGCGGTTGCAGC -3'

Vector, forward primer

5'-CGCTGCTGAAAGCCATCCTGGACAAACTG-3'

Vector, reverse primer

5'-GCCCGGATTTTGCAGTTGTTGCAGTTG-3'

NMR spectra

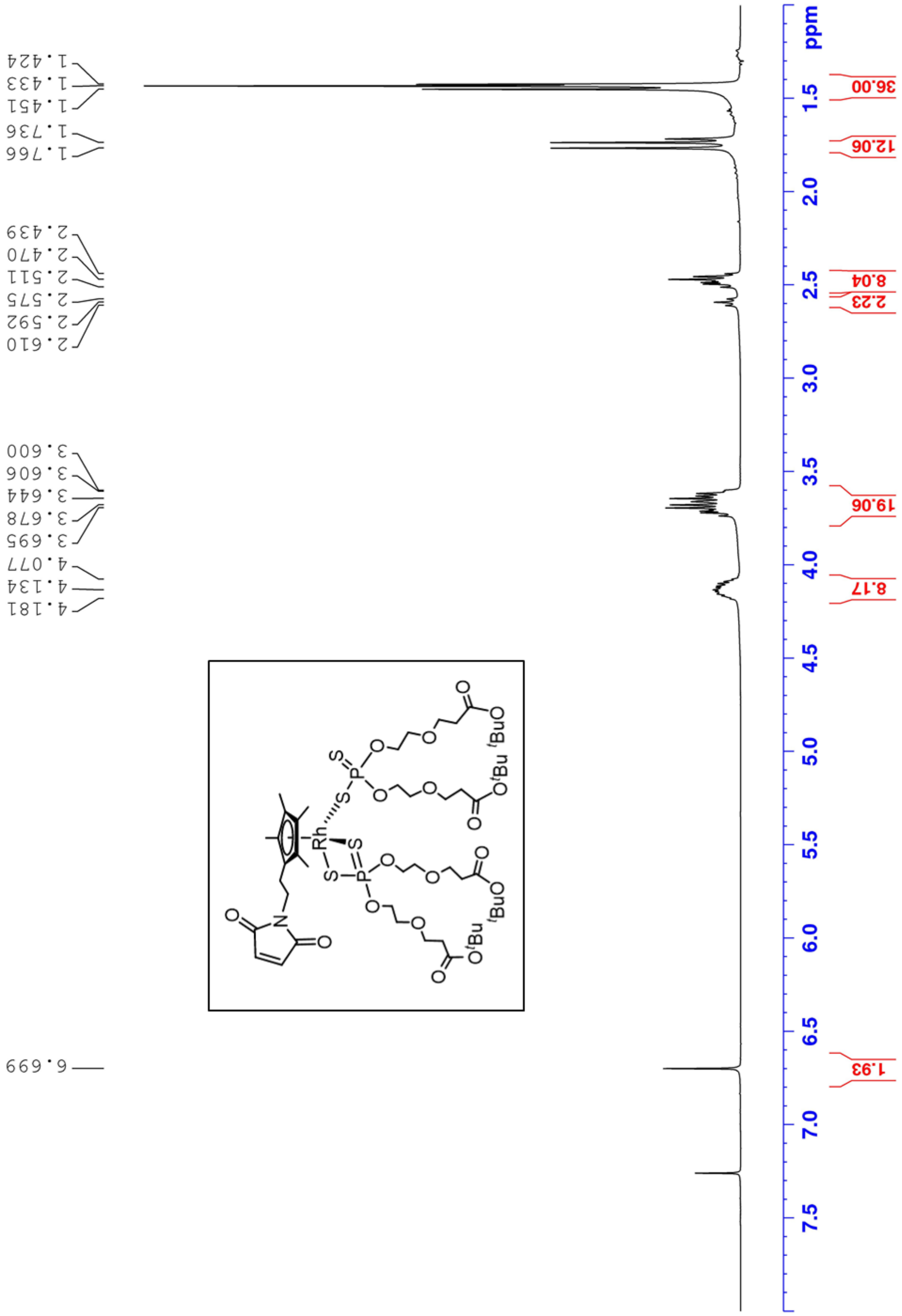

Figure S17. ^{1}H NMR spectrum of **S3** in $CDCl_3$.

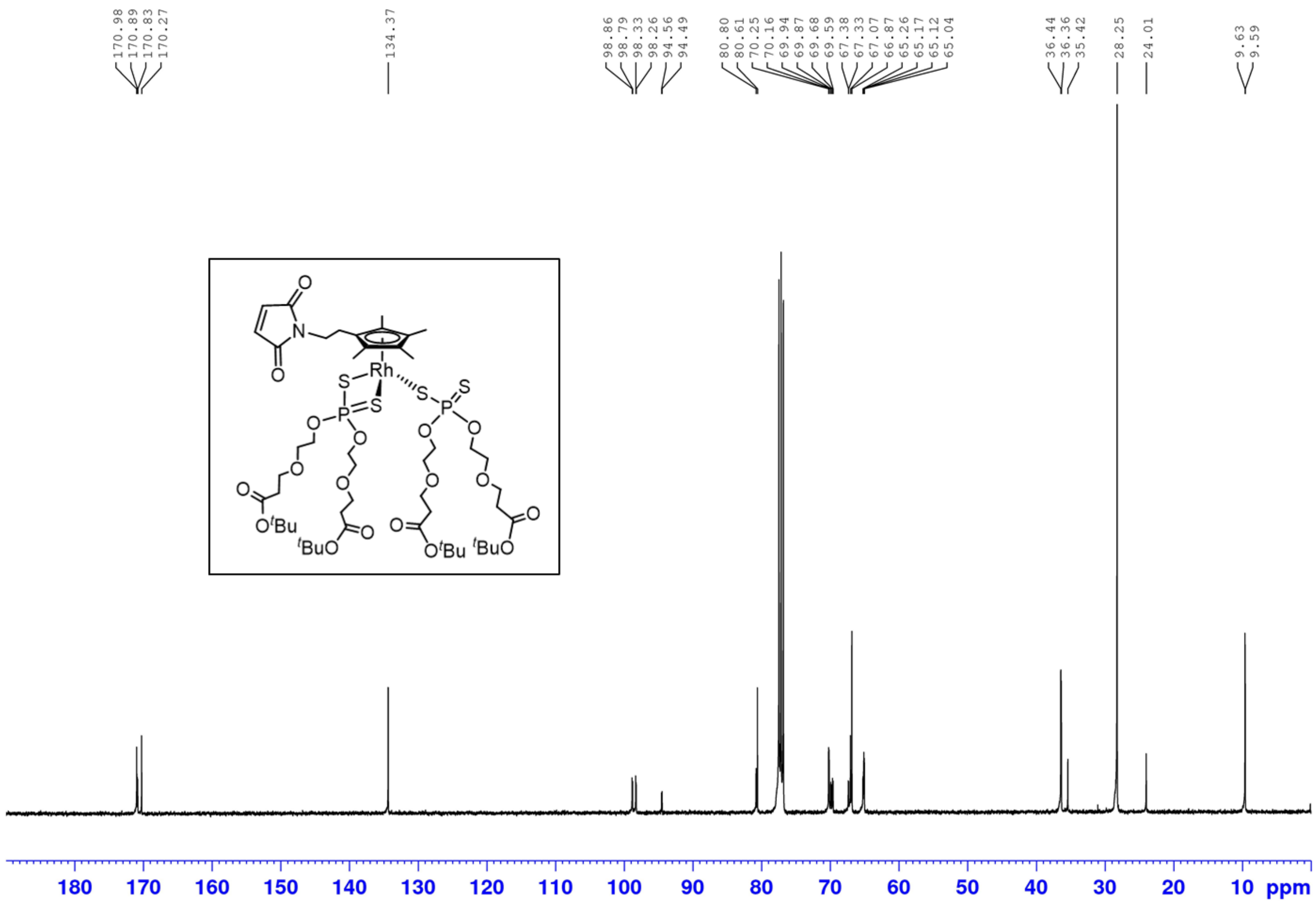

Figure S18. ^{13}C NMR spectrum of **S3** in $CDCl_3$.

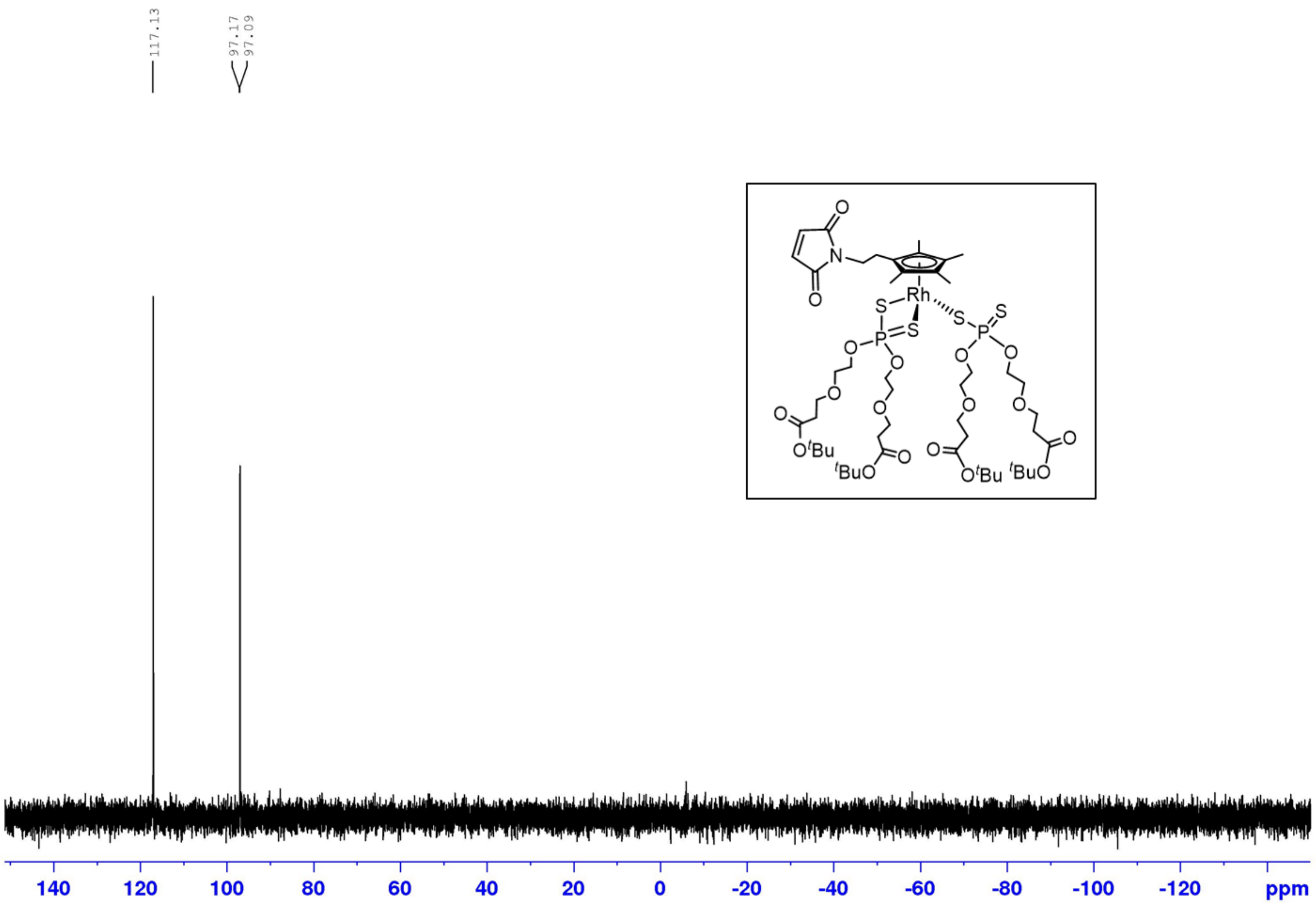

Figure S19. ^{31}P NMR spectrum of **S3** in $CDCl_3$.

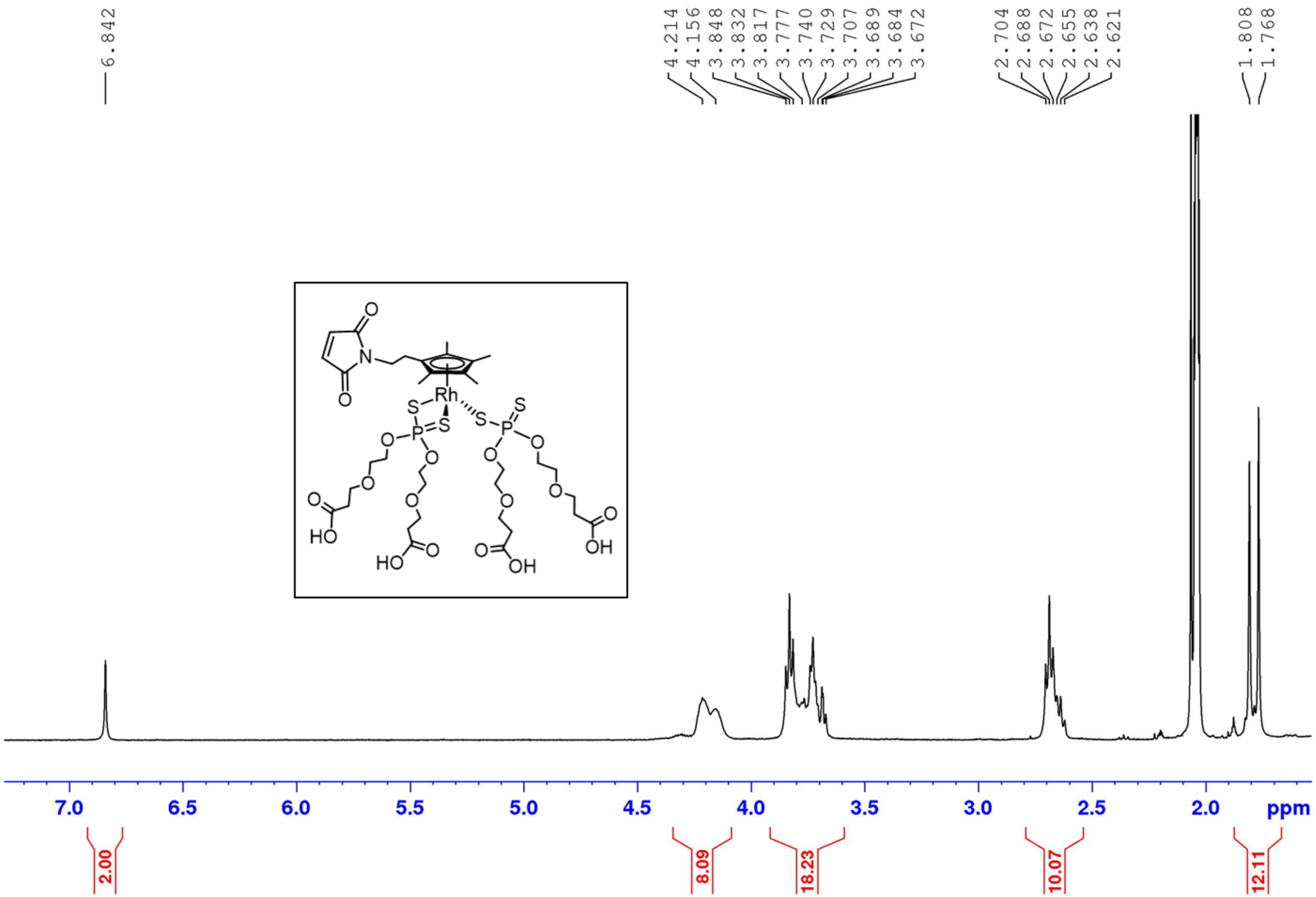

Figure S20. ^{1}H NMR spectrum of **1′** in acetic acid-d_4.

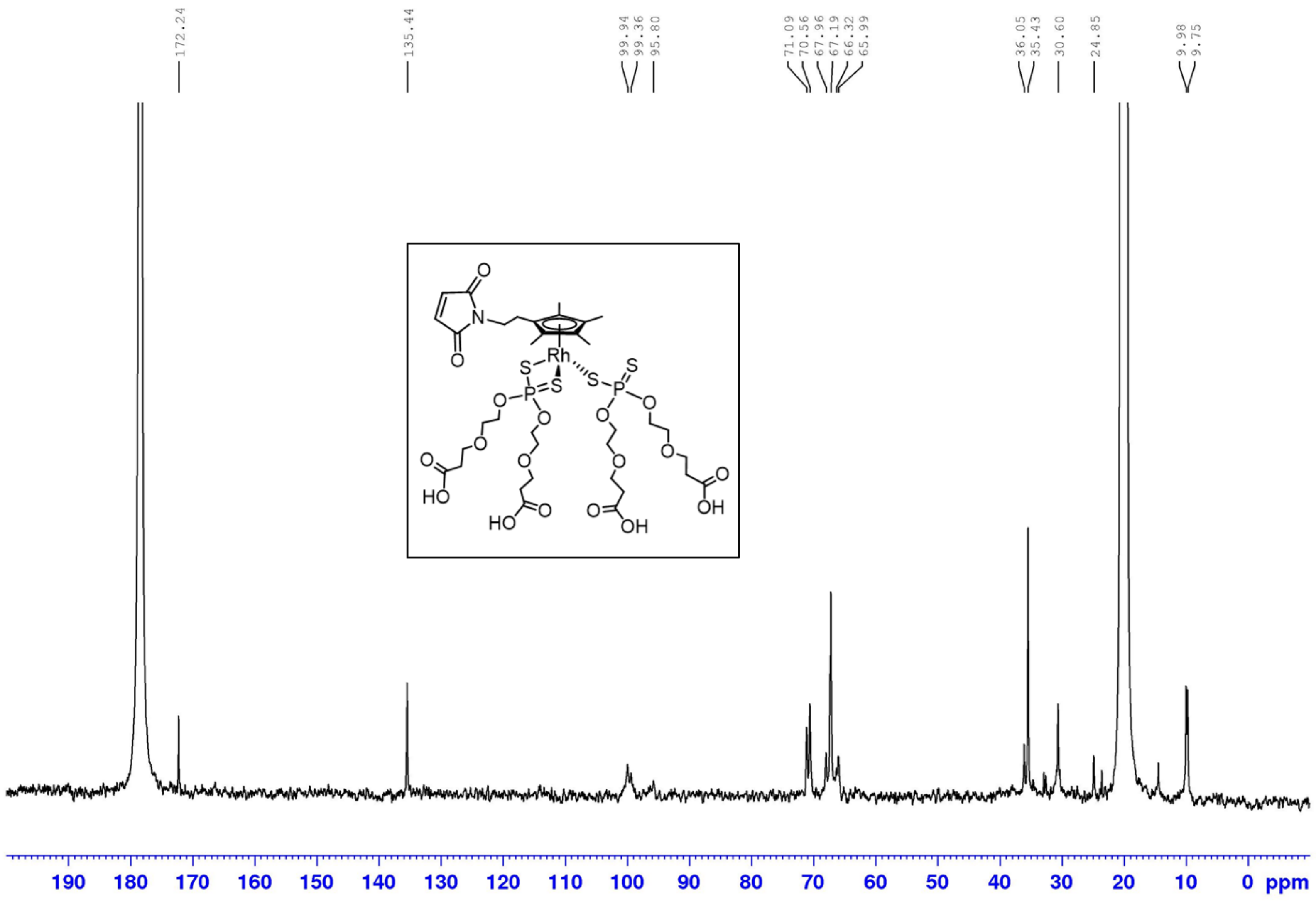

Figure S21. ^{13}C NMR spectrum of **1′** in acetic acid-d_4.

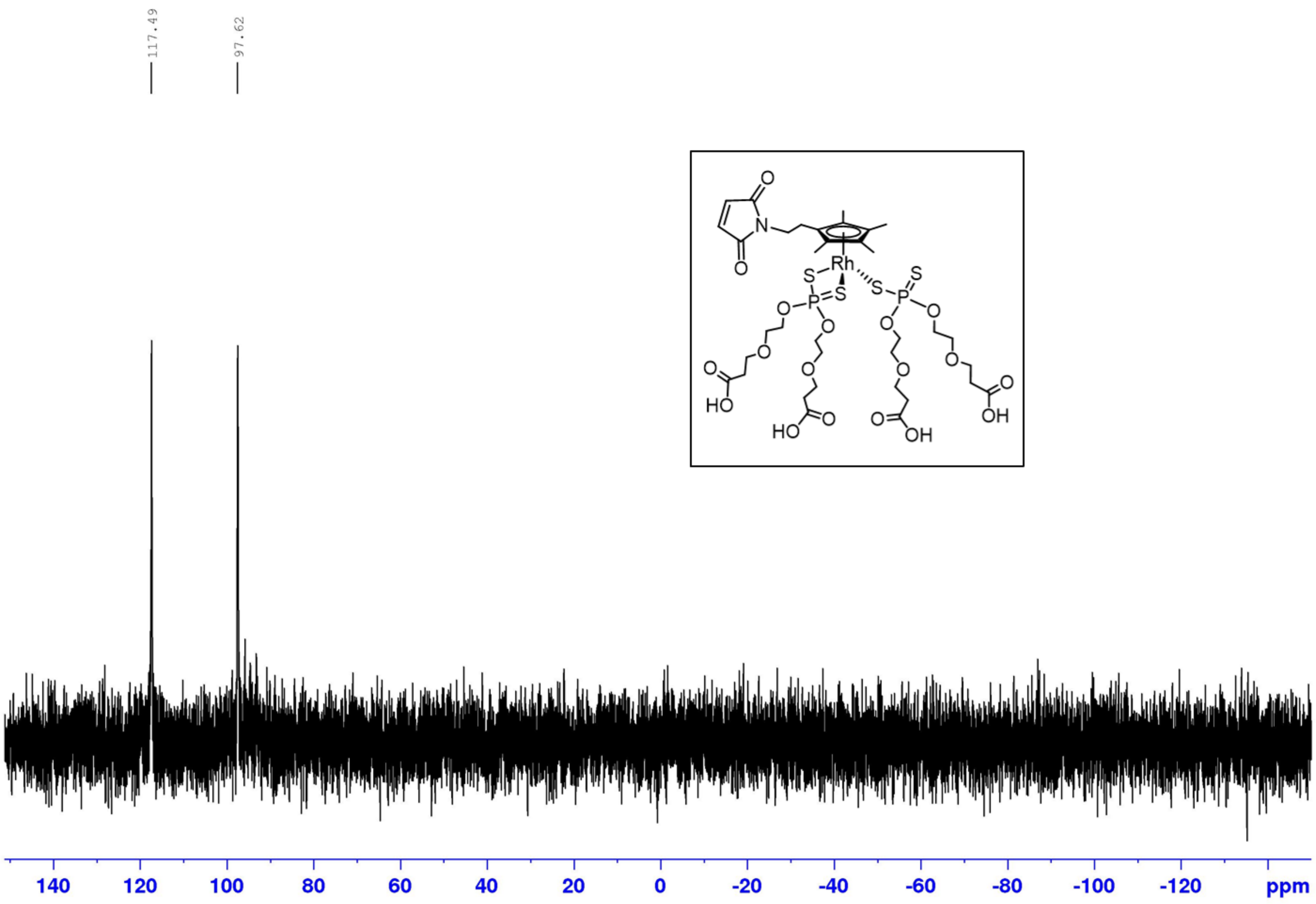

Figure S22. ^{31}P NMR spectrum of **1′** in acetic acid-d_4.

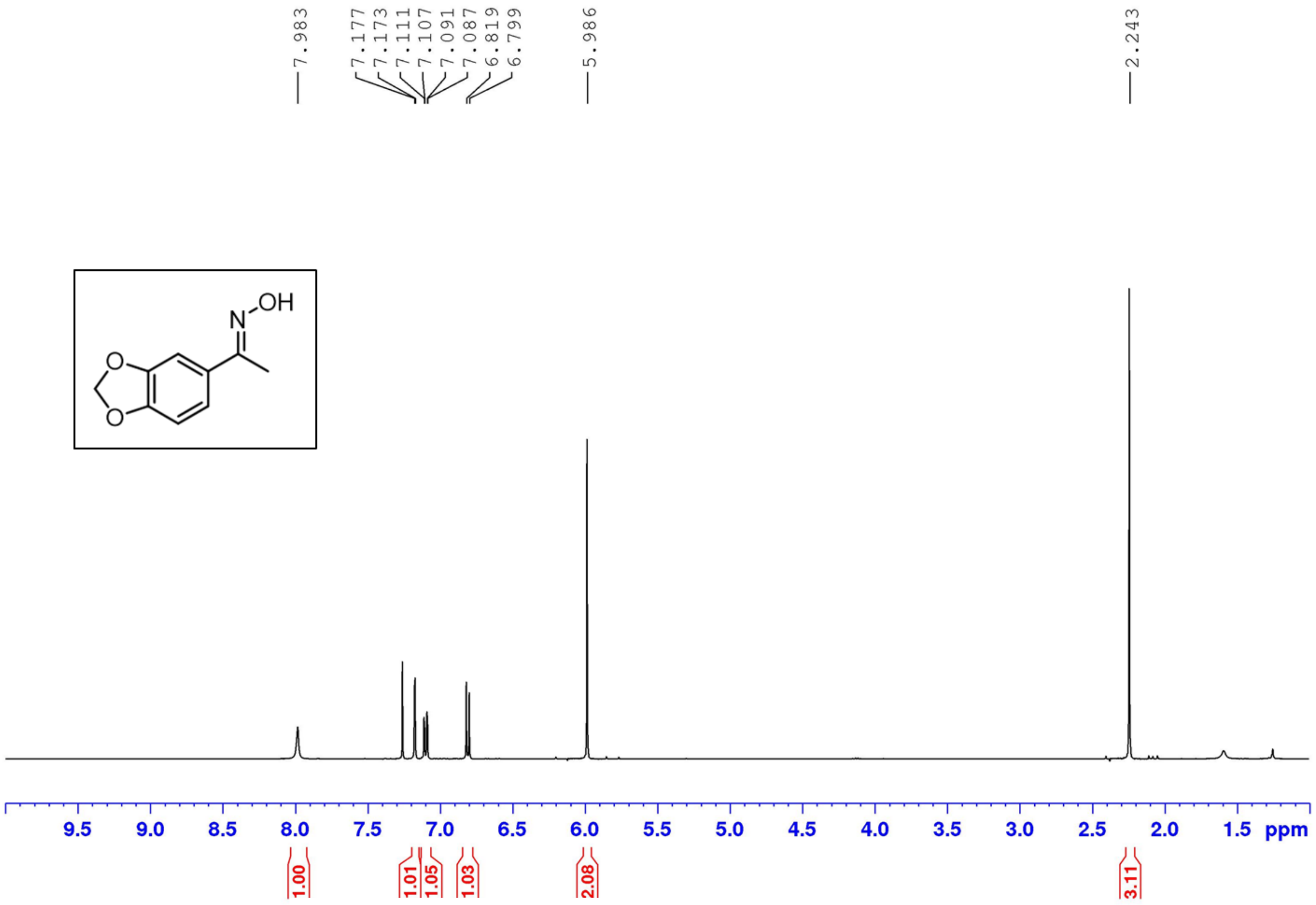

Figure S23. ^{1}H NMR spectrum of **2b** in $CDCl_3$.

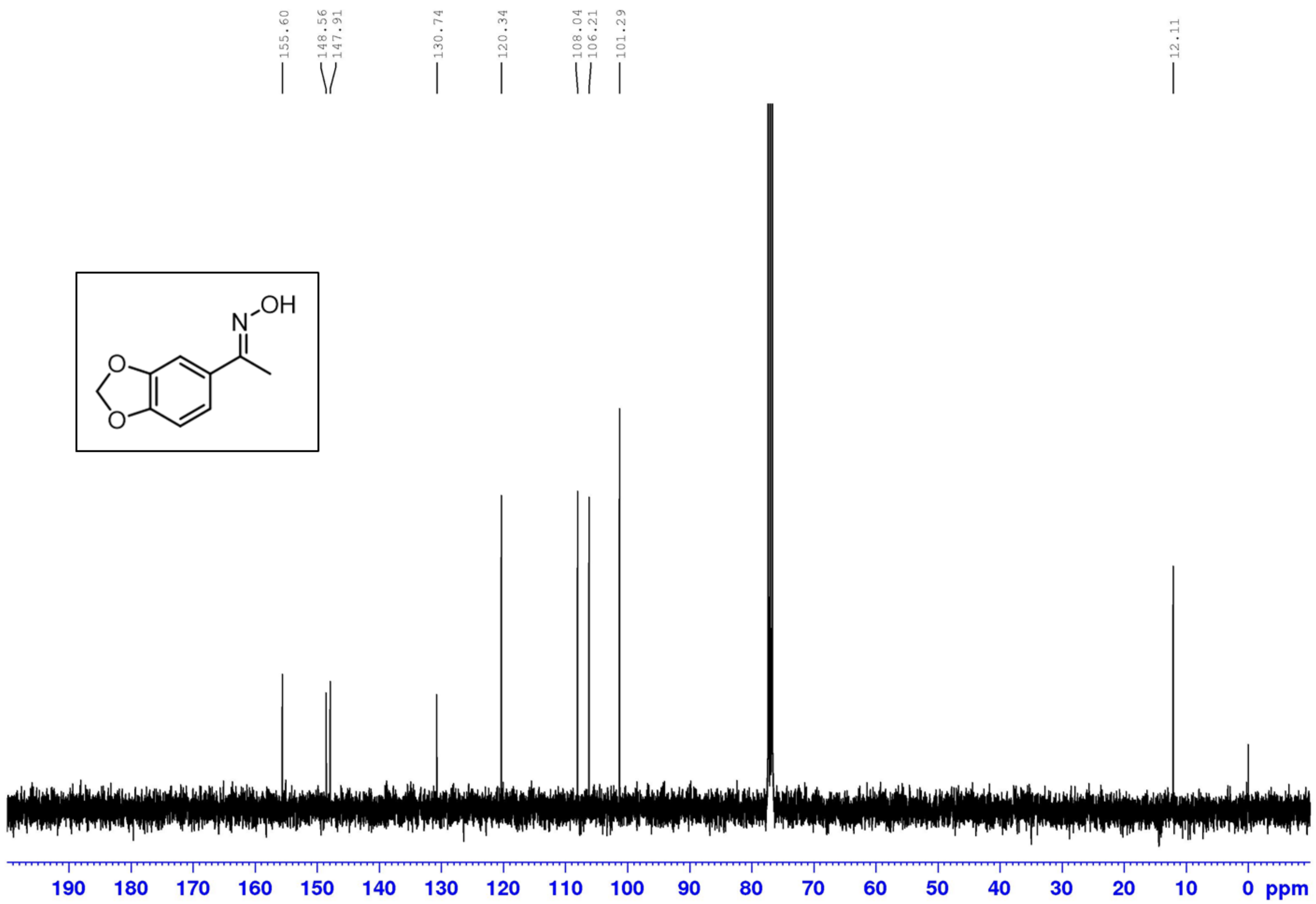

Figure S24. ^{13}C NMR spectrum of **2b** in $CDCl_3$.

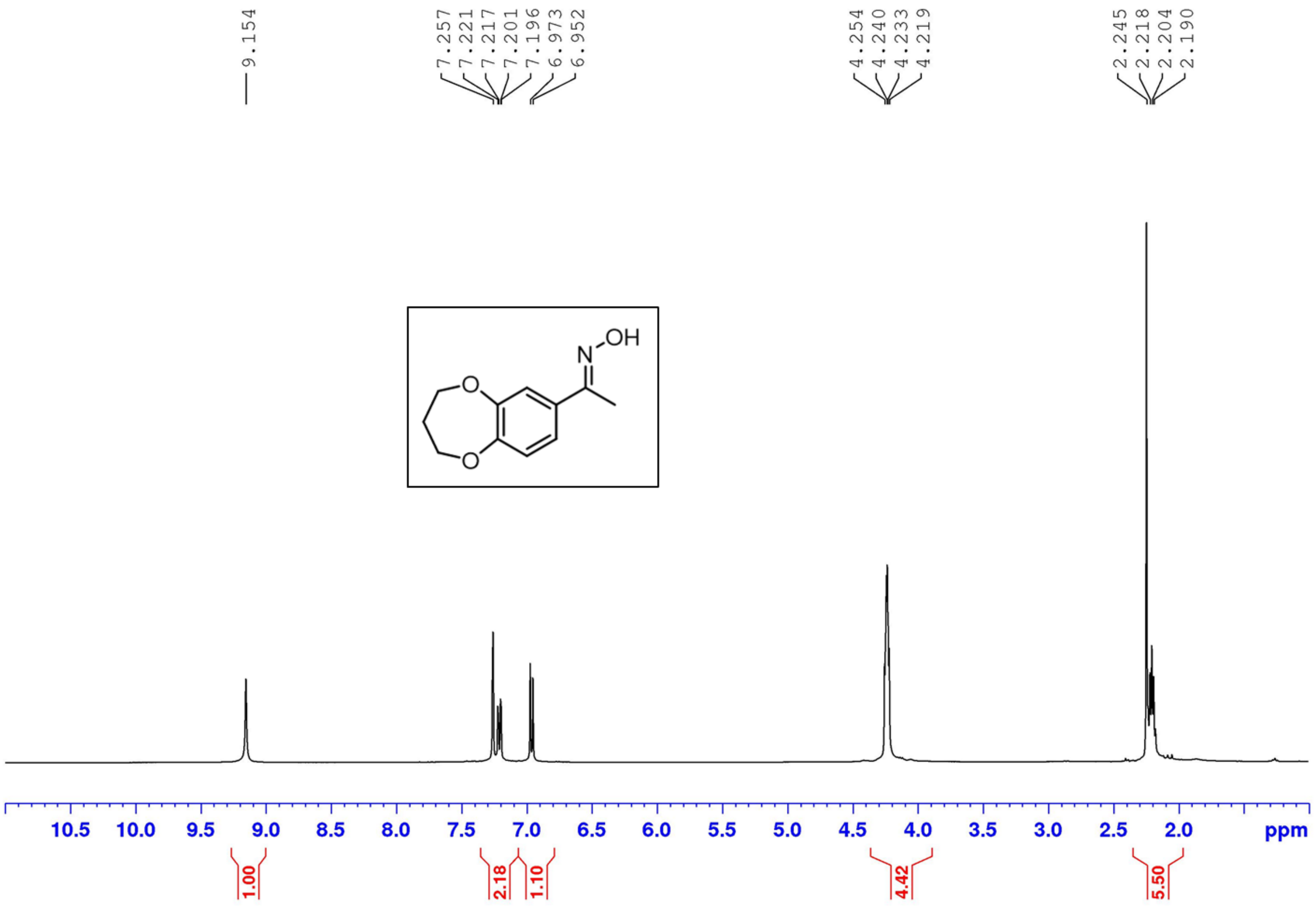

Figure S25. ^{1}H NMR spectrum of **2c** in $CDCl_3$.

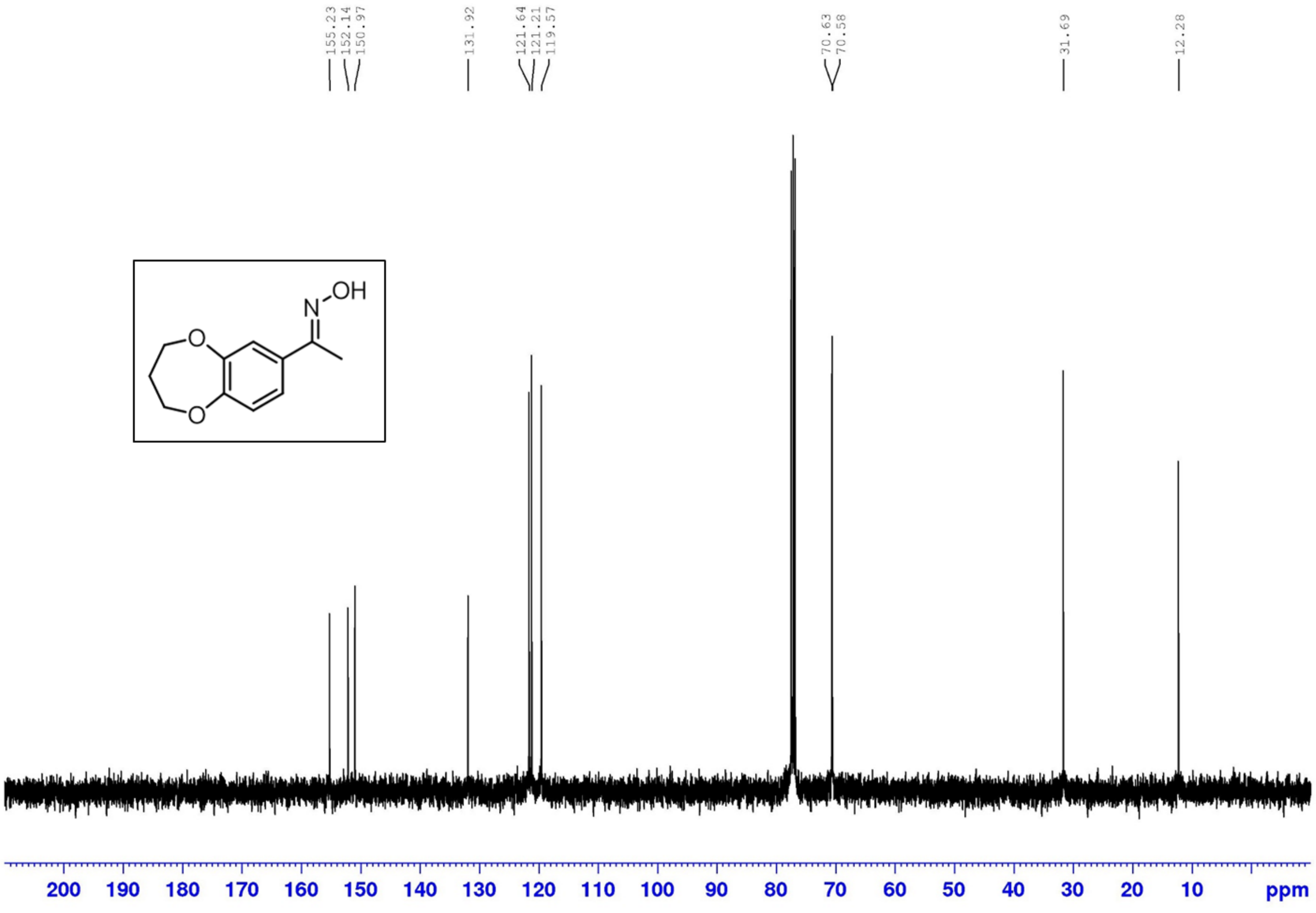

Figure S26. ^{13}C NMR spectrum of **2c** in $CDCl_3$.

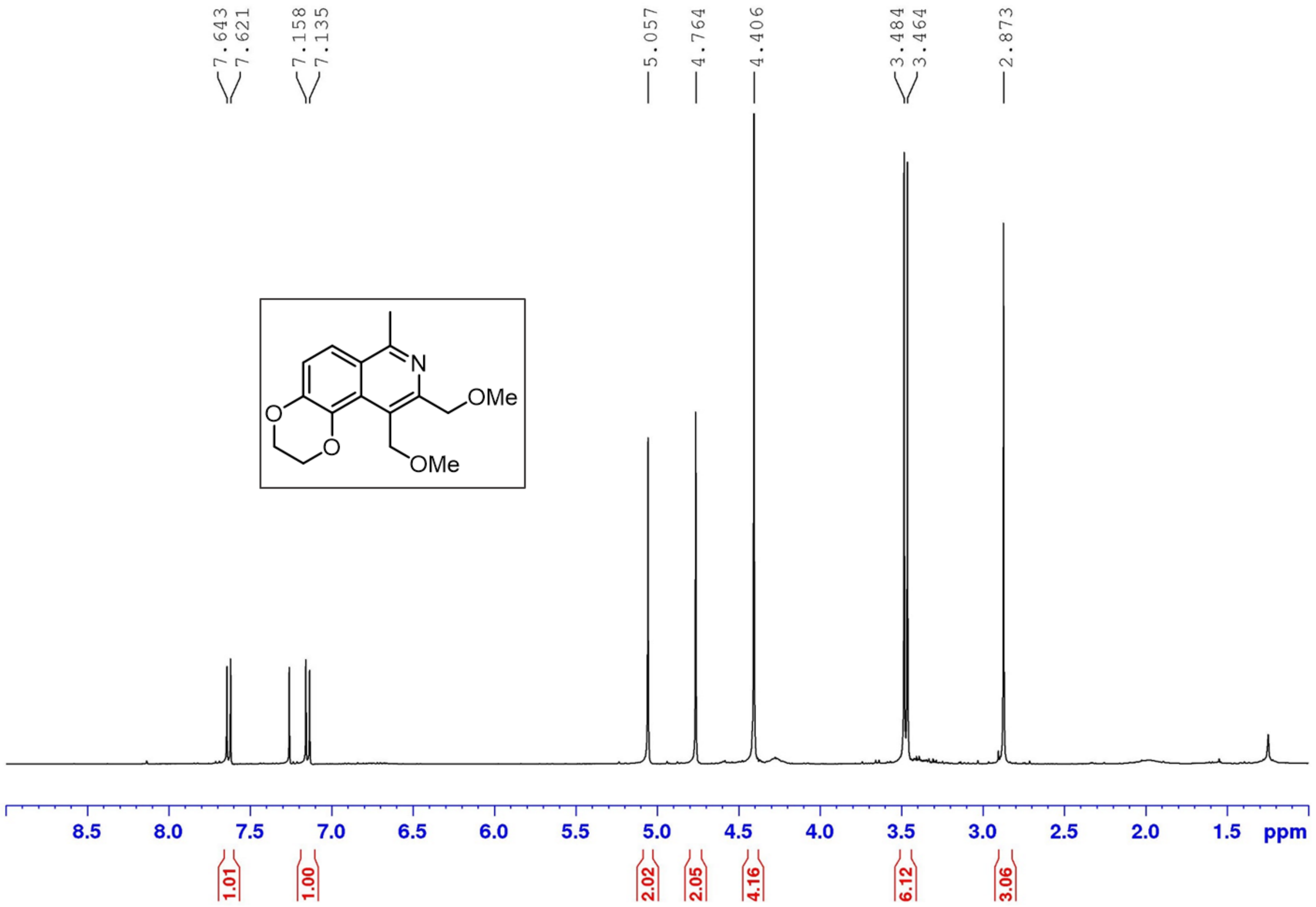

Figure S27. ^{1}H NMR spectrum of **4aa** in $CDCl_3$.

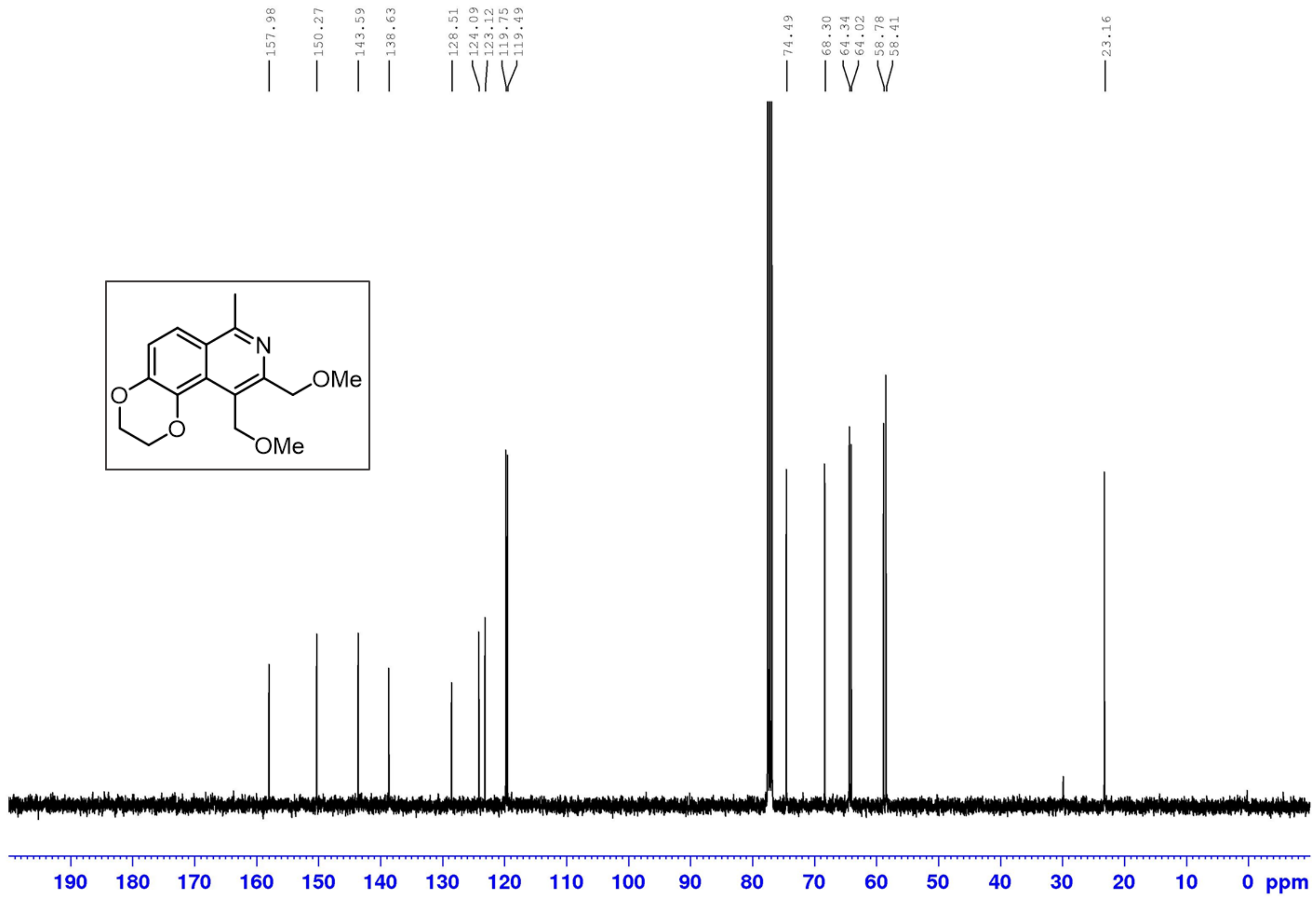

Figure S28. ^{13}C NMR spectrum of **4aa** in $CDCl_3$.

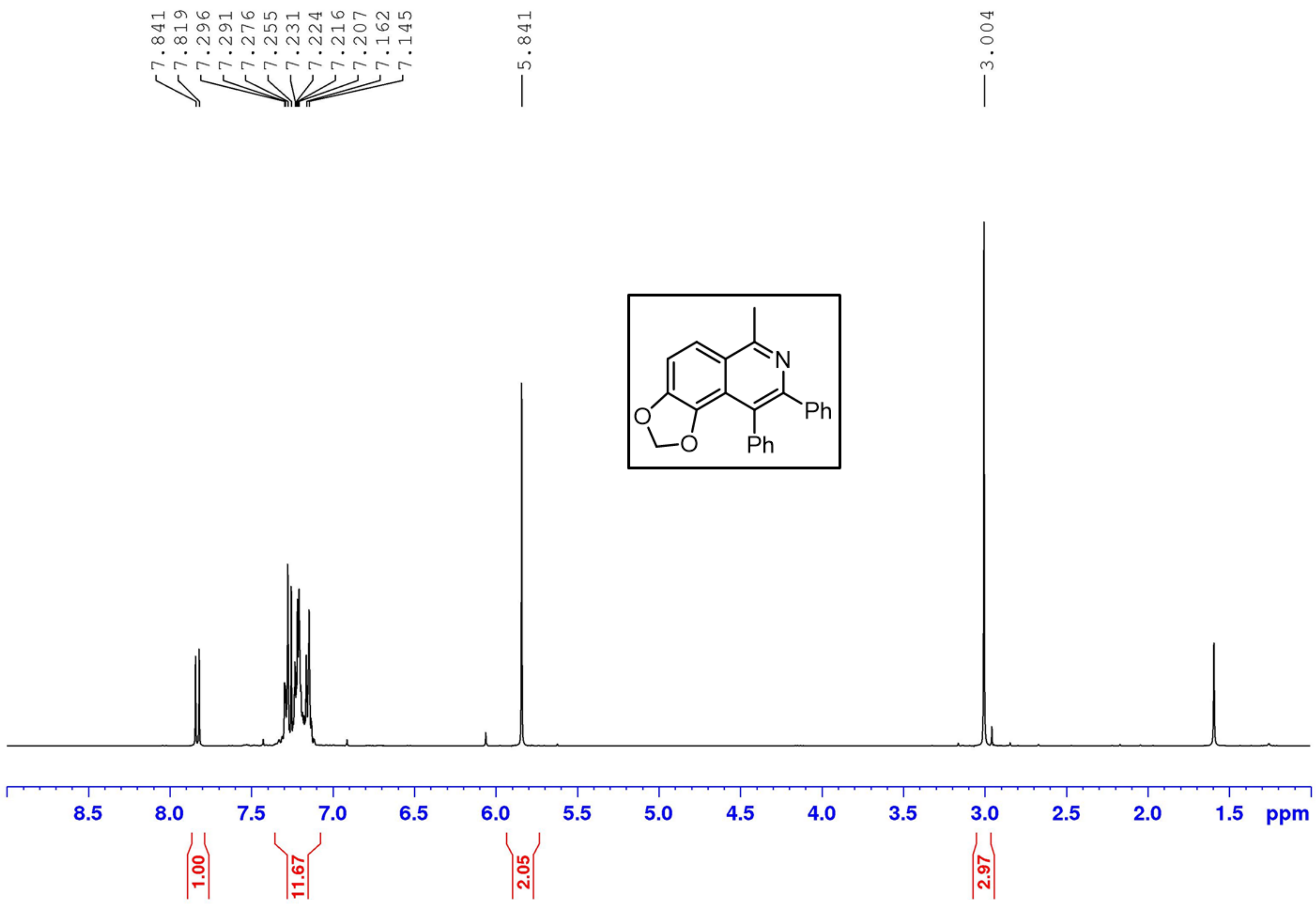

Figure S29. ^{1}H NMR spectrum of **4bb** in $CDCl_3$.

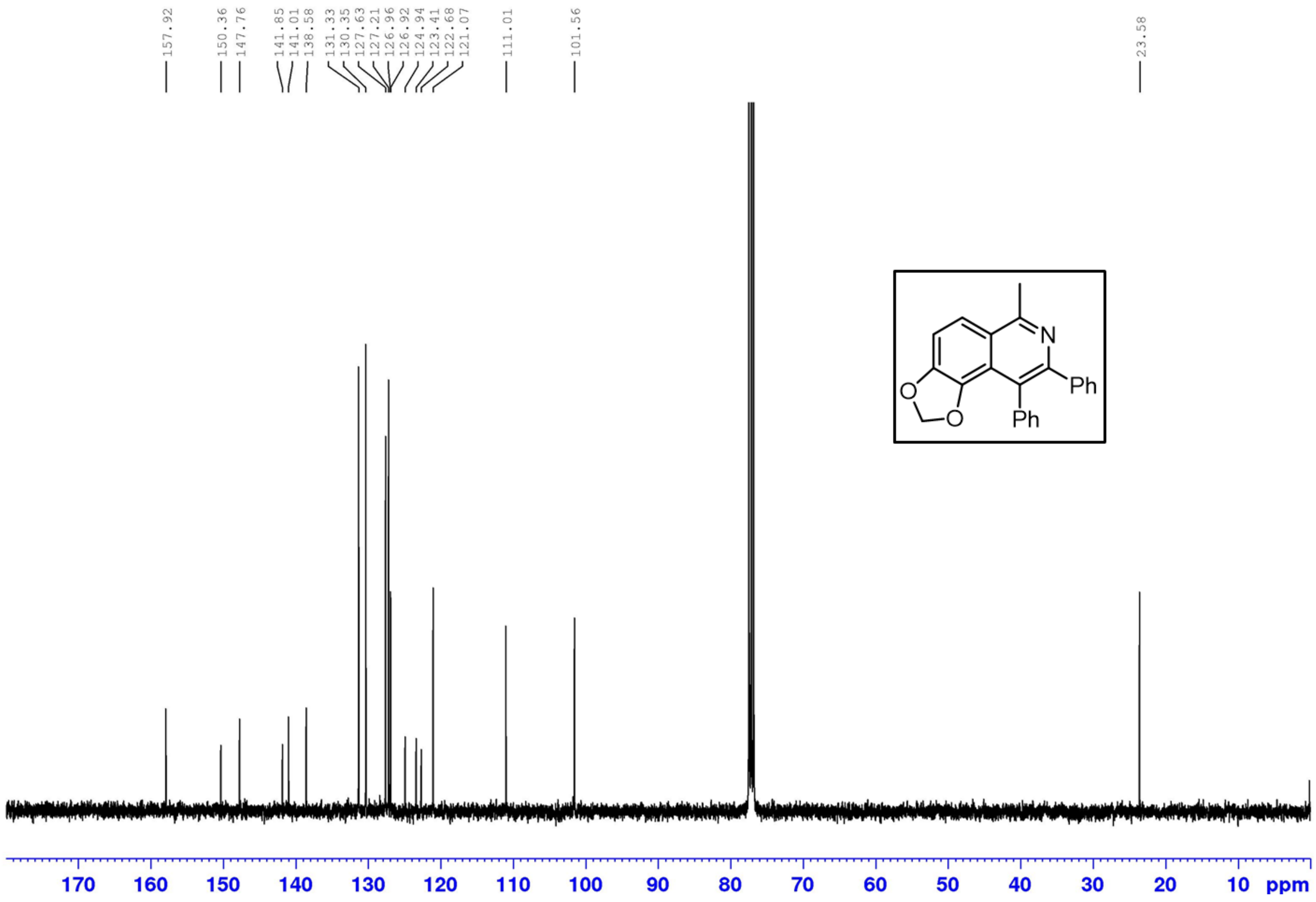

Figure S30. ^{13}C NMR spectrum of **4bb** in $CDCl_3$.

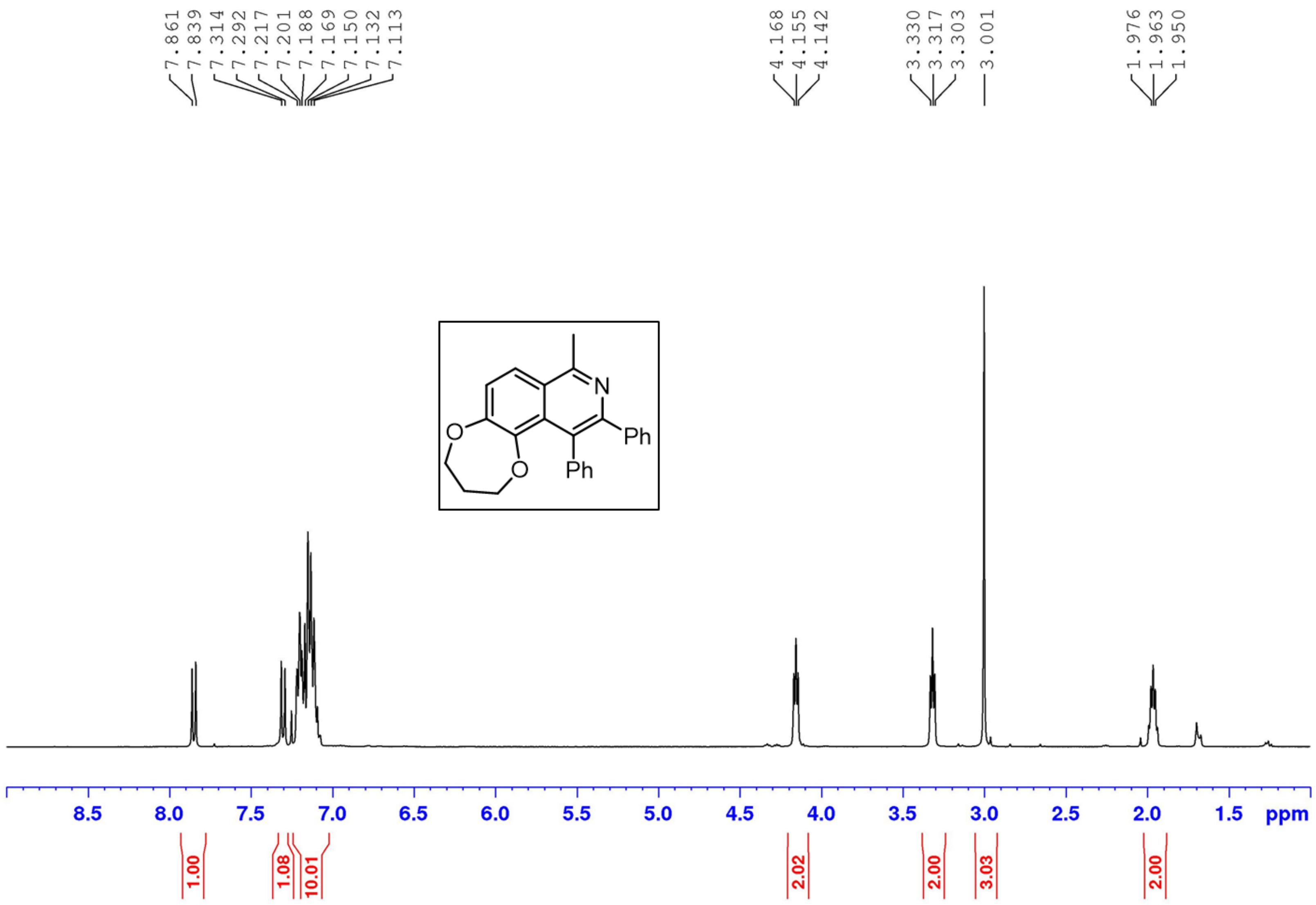

Figure S31. ^{1}H NMR spectrum of **4cb** in $CDCl_3$.

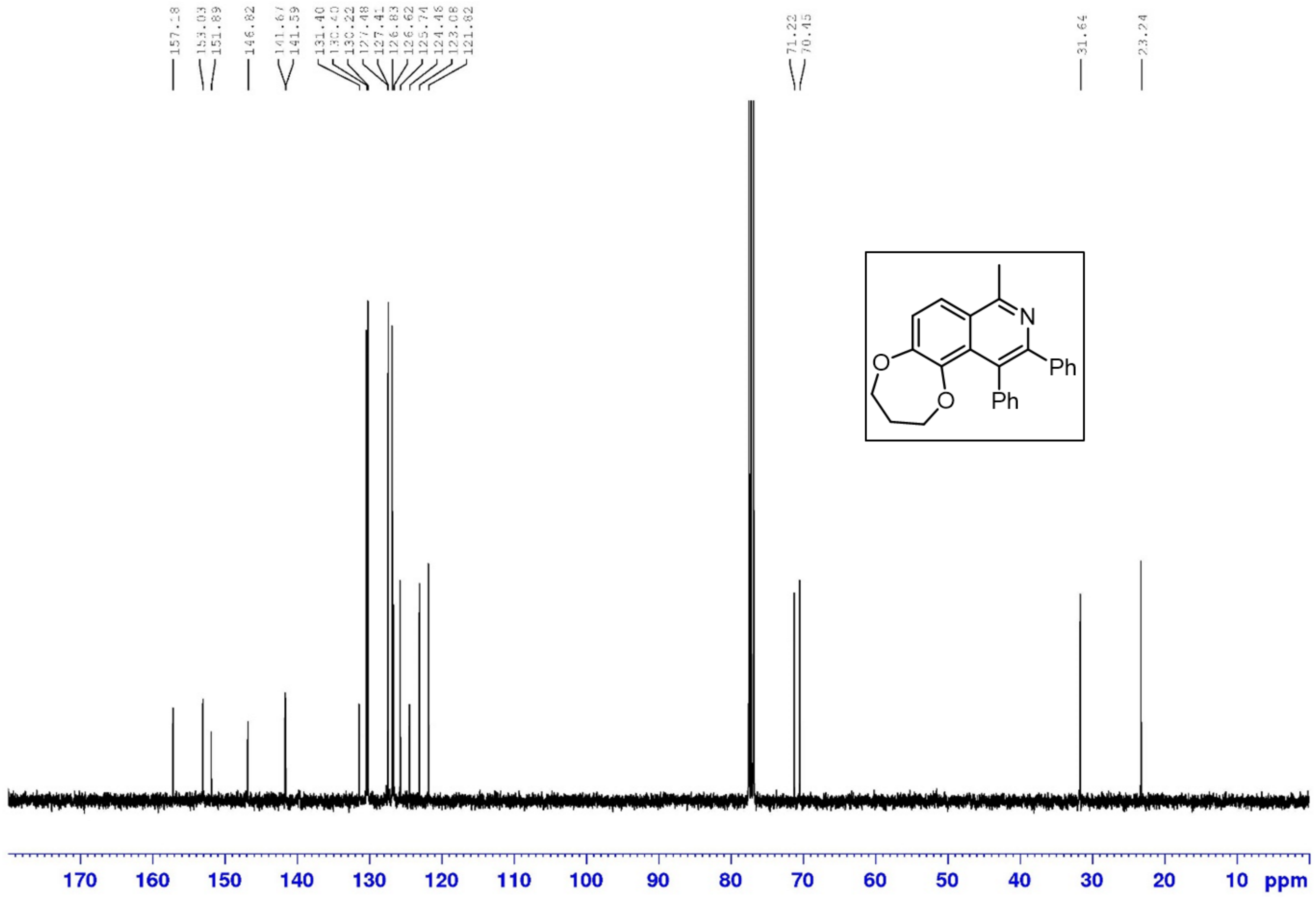

Figure S32. ^{13}C NMR spectrum of **4cb** in $CDCl_3$.

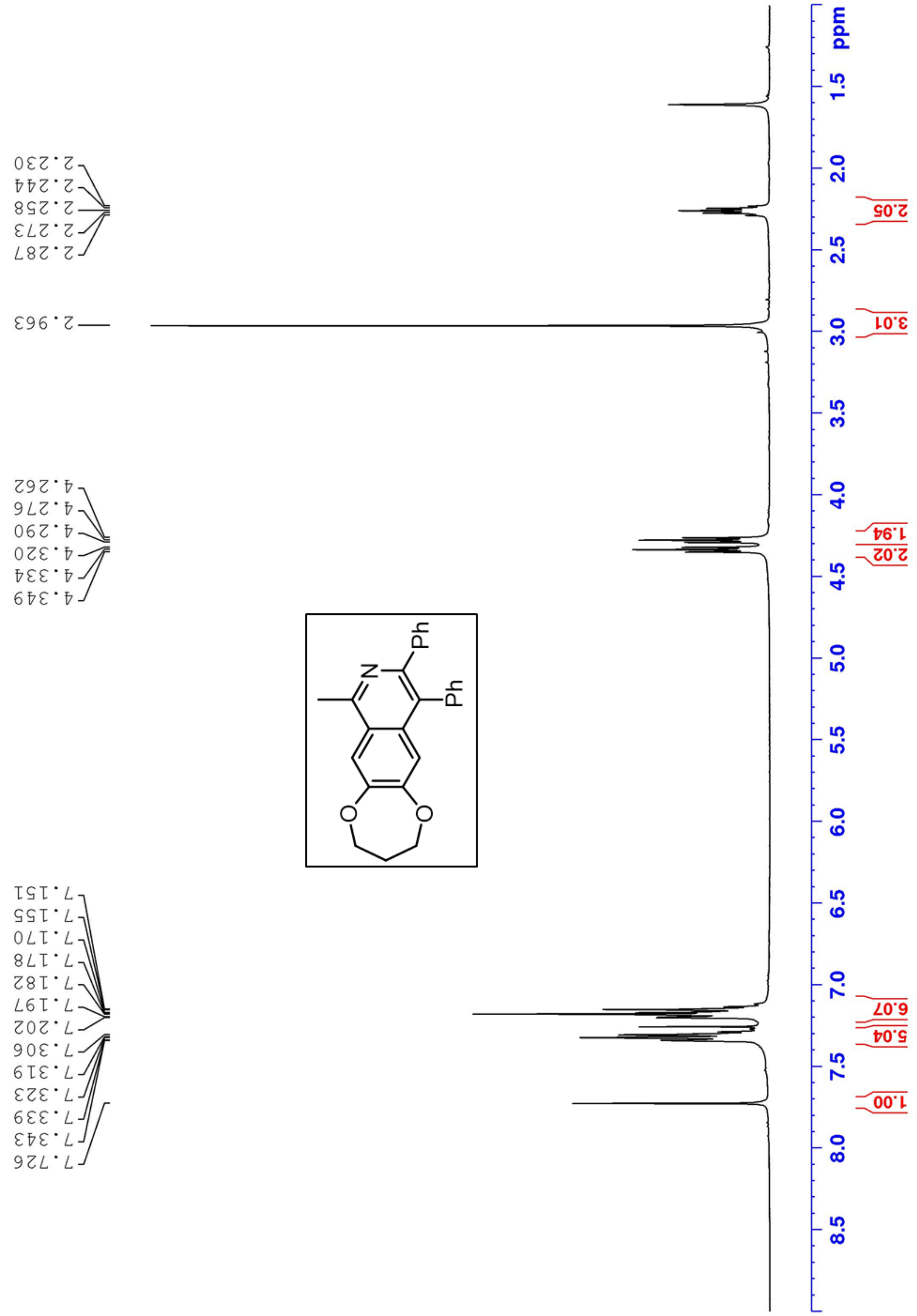

Figure S33. ^{1}H NMR spectrum of **5cb** in $CDCl_3$.

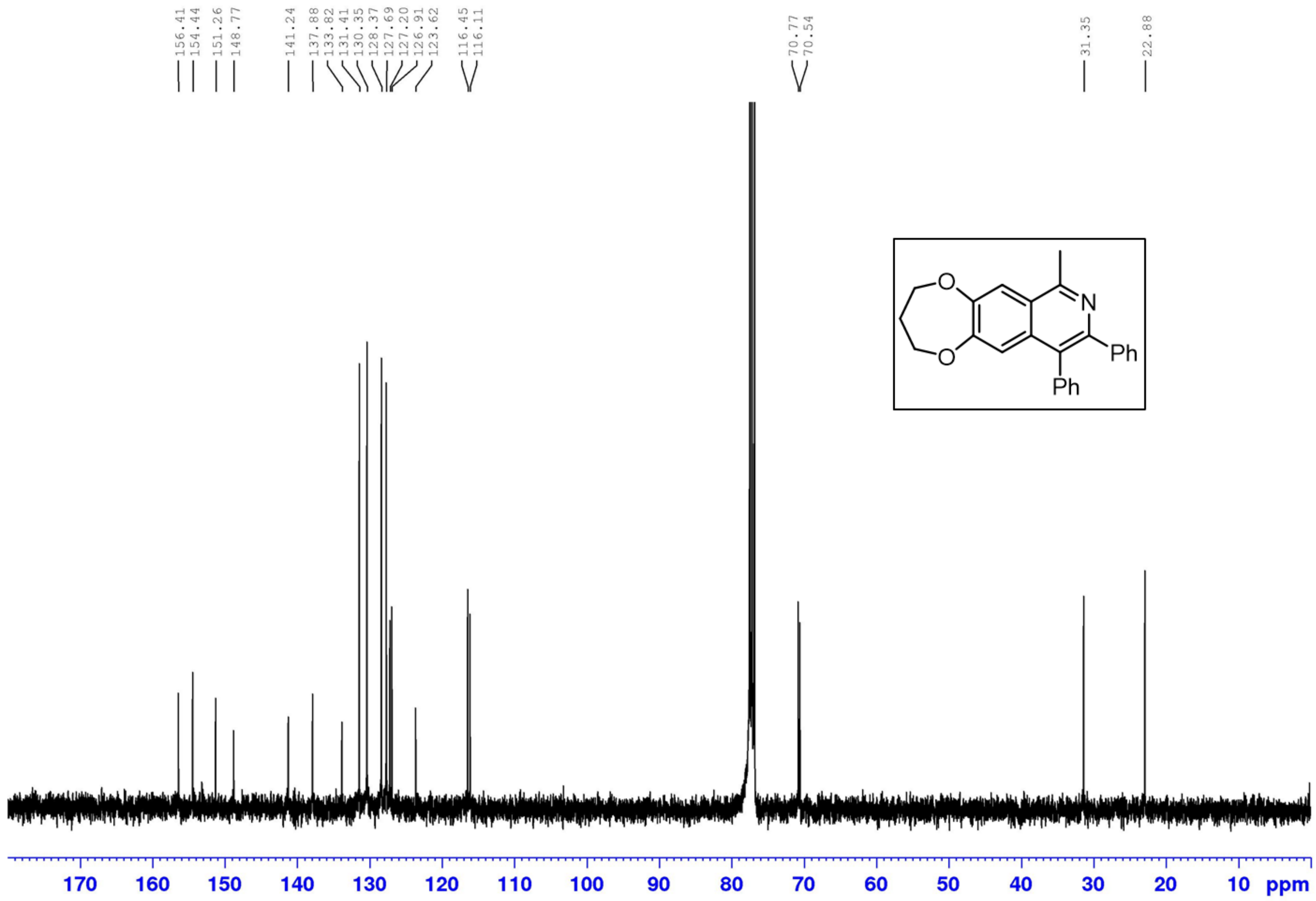

Figure S34. ^{13}C NMR spectrum of **5cb** in $CDCl_3$.

References

[1] S. Kato, A. Onoda, A. R. Grimm, K. Tachikawa, U. Schwaneberg, T. Hayashi, *Inorg. Chem.* **2020**, DOI 10.1021/acs.inorgchem.0c02245

[2] L. Peng, Z. Zhang, C. Lei, S. Li, Z. Zhang, X. Ren, Y. Chang, Y. Zhang, Y. Xu, K. Ding, *ACS Med. Chem. Lett.* **2019**, *10*, 767–772.

[3] T. Reiner, D. Jantke, A. Raba, A. N. Marziale, J. Eppinger, *J. Organomet. Chem.* **2009**, *694*, 1934–1937.

[4] X. Zhang, D. Chen, M. Zhao, J. Zhao, A. Jia, X. Li, *Adv. Synth. Catal.* **2011**, *353*, 719–723.
